# Cheese3D enables sensitive detection and analysis of whole-face movement in mice

**DOI:** 10.1038/s41593-026-02262-8

**Published:** 2026-04-27

**Authors:** Kyle Daruwalla, Irene Nozal Martin, Linghua Zhang, Diana Naglič, Andrew Frankel, Catherine Rasgaitis, Rubin Zhao, Xinyan Zhang, Zainab Ahmad, Jeremy C. Borniger, Xun Helen Hou

**Affiliations:** 1 Cold Spring Harbor Laboratory, Cold Spring Harbor, NY USA; 2 Dept. of Neuroscience, Stony Brook University, Stony Brook, NY USA

**Keywords:** Behavioural methods, Image processing, Hardware and infrastructure, Motor control, Neurophysiology

## Abstract

Facial expressions and movements, from a subtle and ephemeral grimace to vigorous and rapid chewing, offer direct insights into the moment-to-moment changes of neural and physiological processes. Mice, with discernible facial responses and evolutionarily conserved mammalian facial movement control circuits, provide an ideal model in which to unravel the link between facial movement and underlying states. However, existing frameworks lack the spatial or temporal resolution to sensitively track all movements of the mouse face because of its small and conical form factor. We introduce Cheese3D, a computer vision system that captures high-speed 3D motion of the entire mouse face (including ears, eyes, whisker pad and jaw, covering both sides of the face), using a calibrated six-camera array. The interpretable framework extracts dynamics of anatomically meaningful 3D facial features in absolute world units at sub-mm precision. The precise face-wide motion data generated by Cheese3D provides clear insights, as shown by proof-of-principle experiments predicting anesthetic depth from changing facial patterns, inferring tooth and muscle anatomy from fast ingestion motions across the entire face, measuring minute differences in movements evoked by brainstem stimulation and relating neural activity to spontaneous facial movements, including expressive features only measurable in 3D (for example, angles of ear motion). Cheese3D can serve as a discovery tool that renders subtle mouse facial movements as a highly interpretable readout of otherwise hidden processes.

## Abstract (web summary)

The authors developed Cheese3D, a hardware–software framework for precise and sensitive measurement of whole-face movements in mice that enables quantitative inference of neural and physiological processes.

## Main

Facial expressions and movements, from grimacing to chewing, are powerful reflections of health and disease^1,2^. Studying how coordinated motion of individual facial regions gives rise to multi-functional whole-face movements can therefore provide unique insights into physiological processes^3^. Work to date suggests that we can infer pain, distress and sensory input based on subtle facial movement patterns in humans as well as rodents^4–12^. Therefore, once deciphered, the face can serve as a high-bandwidth and compact window into the unseen brain and body condition of animals. Mice share evolutionarily conserved facial movement control circuits with other mammals, including humans. Facial muscles controlling eyes, ears, whiskers, nose and mouth receive direct commands from motor nuclei in the brainstem, instead of in the spinal cord, and are thus positioned relatively close to processing centers in the brain^13–15^. This shared circuit architecture makes laboratory mice ideally suited to serve as a model system for studying the link between facial movements and brain and body processes.

**Fig. 1:**
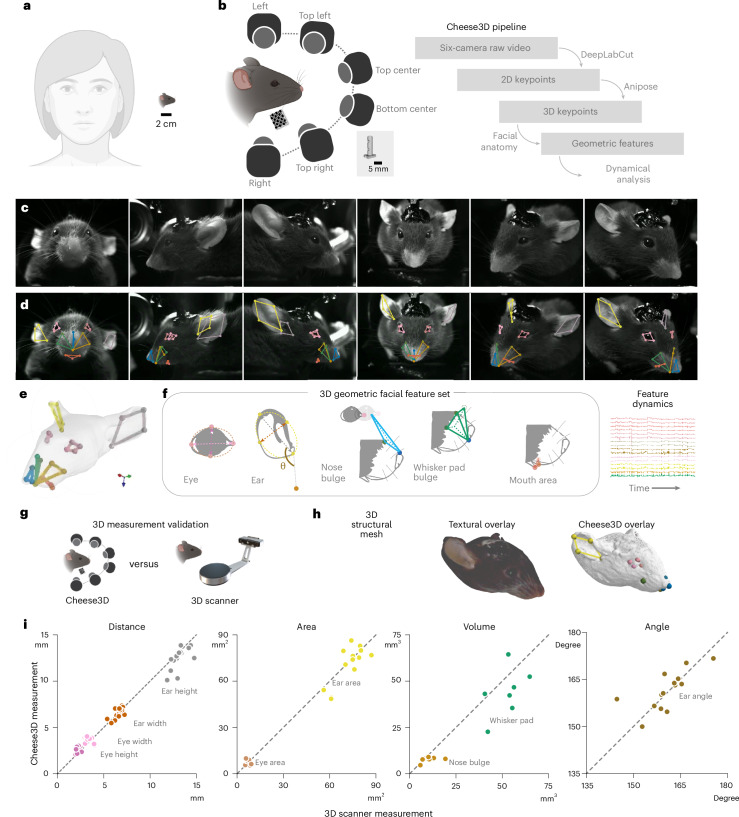
Framework and validation of capturing face-wide movement as 3D geometric features in mice. **a**, The form factor of the mouse face poses technical challenges to track face-wide movement compared to existing technology tailored for the human face. Created in BioRender; CSHL, T. H. L. https://BioRender.com/6sr5jnd (2026). **b**, Schematic of the hardware and software framework. Left, the six-camera facial movement capture setup. The ChArUco board shown below the mouse is required for camera calibration. Inset: a headpost designed to image the mouse face without occluding any facial features (see Extended Data Fig. 1b for details). Right, the analysis pipeline, which inputs six-camera raw video and outputs the dynamics of a geometric facial feature set (see Extended Data Fig. 1a for details). Created in BioRender; CSHL, T. H. L. https://BioRender.com/6sr5jnd (2026). **c**, Example synchronized frames from the six-camera setup. **d**, 3D facial keypoints visualized as projections onto the frames shown in **c**. **e**, 3D facial keypoints overlaid onto a 3D template mouse face from a previous publication^55^ used purely as a visual aid. **f**, Output of Cheese3D. Left, illustrations of the set of anatomically based facial features, including 3D distances, areas, volumes and angles across facial regions (see text and methods for details). Right, example time series of the 3D feature set. Created in BioRender; CSHL, T. H. L. https://BioRender.com/6sr5jnd (2026). **g**, Experimental design to validate Cheese3D facial feature measurement (anesthetized) compared to 3D scanner. Created in BioRender; CSHL, T. H. L. https://BioRender.com/6sr5jnd (2026). **h**, Example mouse face 3D mesh obtained with a 3D scanner. Left, with texture overlay, showing fur and color details. Right, the same mesh overlaid with 3D keypoints obtained from Cheese3D to compare the two. **i**, Comparison of Cheese3D facial feature measurement with 3D scanner data, grouped by distances, areas, volumes and angles, from left to right. Each dot represents one animal for midline facial features (nose bulge, whisker pad bulge) and one of the two structures for one animal (*n* = 7 mice). Measurements for lateralized facial features (eyes, ears) contain both left and right sides and thus have twice the amount of data points compared to midline features (nose bulge, whisker pad bulge). The mouth area is the only feature excluded from the comparison, given that it cannot be reliably measured on the 3D scanner because of the orientation of the mouse face relative to the projector. Means ± root mean squared errors (RMSE) are as follows: eye height, 2.62 ± 0.52 mm; eye width, 3.71 ± 0.63 mm; ear height, 12.45 ± 1.13 mm; ear width, 6.54 ± 0.43 mm; ear angle, 161.45 ± 4.86^∘^; eye area, 8.14 ± 2.27 mm^2^; ear area, 71.18 ± 7.39 mm^2^; nose bulge, 7.74 ± 4.75 mm^3^; whisker pad bulge, 43.87 ± 13.57 mm^3^. Source data.

Realizing the potential of the face as an informative readout requires a framework with the sensitivity, precision and accuracy to quantitatively relate facial movement to internal state. Although recent advances in computer vision have fueled state-of-the-art methods for human facial movement tracking^16,17^, similar approaches to characterizing face-wide movements in mice encounter unique technical challenges. Mouse faces are an order of magnitude smaller than human faces, and the conical shape of their head makes it difficult to capture face-wide movement using a single camera (Fig. 1a). Existing methods rely on zooming in to the motion of a single facial region (for example, whiskers, tongue) or a subset of facial regions on one side of the face^3,18,19^. Alternative methods have discarded temporal dynamics by focusing on still images of the face^6,20^. Recent 3D methods hold promise to capture movements of the whole animal^21–24^, but the approach has not been evaluated at the high resolution required to examine the face of a mouse, in which movements are much smaller relative to the full face.

**Extended Data Fig. 1:**
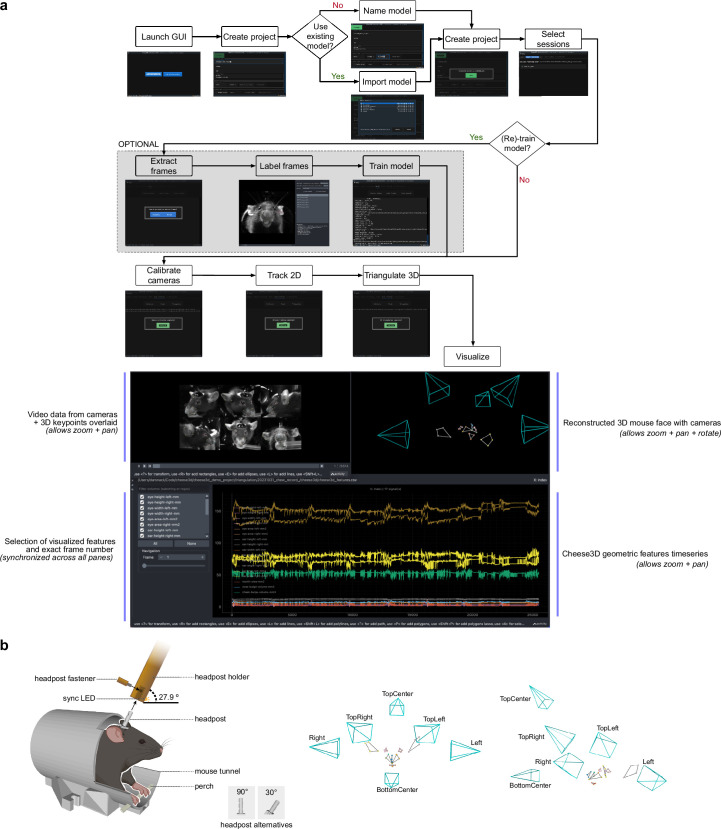
Cheese3D hardware and software pipeline. (**a**)Flowchart of software pipeline, including an interactive data visualizer that shows input video data, tracked keypoints in 3D, and output anatomical features, concurrently. See software documentation for details. (**b**)Hardware schematics. *Left:* Cheese3D headpost angle can be customized to allow access to different brain regions; for example, the 30^∘^ headpost shown here was used for brainstem recording and stimulation shown in Fig. 5. *Right:* Frontal and side view diagram of the camera locations and Cheese3D keypoints, connected by facial region. Created in BioRender. CSHL, T. H. L. (2026) https://BioRender.com/6sr5jnd.

Adapting components from existing markerless pose estimation tools, we carefully designed a hardware and software pipeline (Extended Data Fig. 1a) to create a unified 3D view of the mouse face and introduced technical advancements to reduce keypoint jitters common to existing tools, a critical step to increasing the resolution and sensitivity necessary to study subtle and rapid mouse facial movements. Our approach, Cheese3D, is delivered as an easy-to-use Python software package, and it includes an interactive visualization tool to view the input video data, tracked 3D points on the face and output anatomical features concurrently (Supplementary Video 1). Cheese3D balances the trade-offs in current tools, resulting in the first method sensitive enough to characterize how physiological, cognitive and emotional states drive overt dynamics of the face. Cheese3D captures the whole face—including insightful features such as the ears and both sides of the face, which are absent in existing tools owing to technical challenges—and a wide dynamic range of movements, revealing the potential of facial movement as a noninvasive, information-dense readout of unseen processes.

## Results

### Cheese3D captures robust 3D whole-face movement in mice

The Cheese3D pipeline captures and analyzes synchronous movement of the entire mouse face at 100 Hz temporal resolution. The three pairs of high-speed video cameras (six total) are positioned compactly to capture the frontal view (top center and bottom center cameras), the profile view (left and right cameras) and an elevated half-profile view (top left and top right cameras) (Fig. 1b and Extended Data Fig. 1b). To acquire high-resolution facial video while maintaining comfort for natural behavior, mice are acclimated to sitting in a tunnel with the head secured using a lightweight headpost, custom-designed to allow unobstructed viewing of all facial areas (Fig. 1b inset and Extended Data Fig. 1b). Individual views from the six-camera array are temporally synchronized, and spatial alignment between views is captured through ChArUco calibration^25^. We identified a set of 27 facial keypoints that covers all facial areas in C57BL/6J mice (Fig. 1c–e and Supplementary Video 2; Created in BioRender; CSHL, T. H. L. https://BioRender.com/6sr5jnd (2026)). Each keypoint is in sharp focus and visible by at least two cameras (see Supplementary Table 1) and is reproducibly labeled by different researchers following written guidelines. The calibrated hardware setup and labeling protocol enables us to adapt existing markerless pose estimation techniques—Anipose^22^ and DeepLabCut (DLC)^23^—to create a unified 3D view of the whole mouse face at the spatial and temporal resolutions necessary to study facial movements.

As facial movement is inherently constrained in 3D space, existing 2D methods for mouse facial analysis either require a single camera view, limiting the type of movement studied to those that can be captured in a single plane, or rely on principal component analysis or hidden Markov models to integrate keypoints across multiple uncalibrated views, hindering direct interpretation^3^. Moving from 2D to 3D calibrated face space is critical to enable interpretable feature selection that is physically grounded and verifiable in world units: we selected a set of 17 3D geometric features—distances, angles, areas and volumes in 3D space—constructed from shapes defined by facial keypoints (Fig. 1f). Furthermore, the features are localized to facial regions based upon known muscular anatomy and descriptors of rodent facial movements^5,26^.

**Extended Data Fig. 2:**
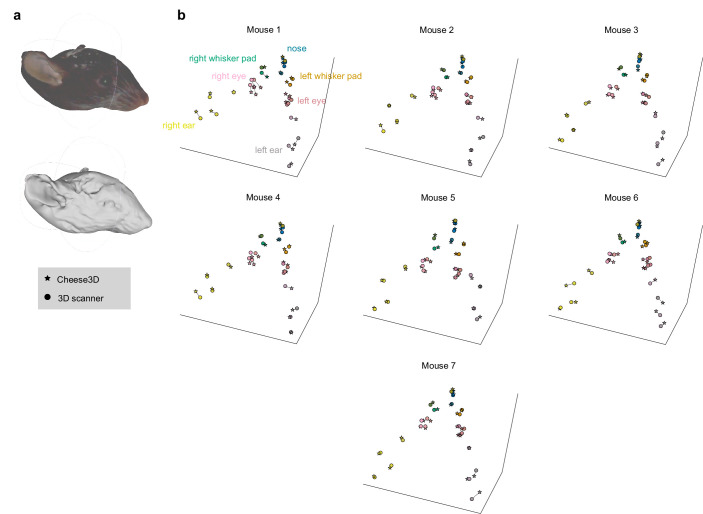
3D facial keypoint comparison between Cheese3D and 3D scanner. (**a**)Example textured (above) and untextured (below) mesh of mouse face obtained in 3D scanner used for validating Cheese3D keypoint placement. (**b**)3D scatter plot showing corresponding 3D scanner (solid circle) and Cheese3D (star) triangulated keypoints for all mice shown in Fig. 1i. Source data.

We evaluated the accuracy of Cheese3D by comparing its resulting 3D geometric features with those measured statically using a 3D scanner (resolution, 50 μm) for the same mouse (Fig. 1g–i, Extended Data Fig. 2 and Supplementary Video 3).

**Extended Data Fig. 3:**
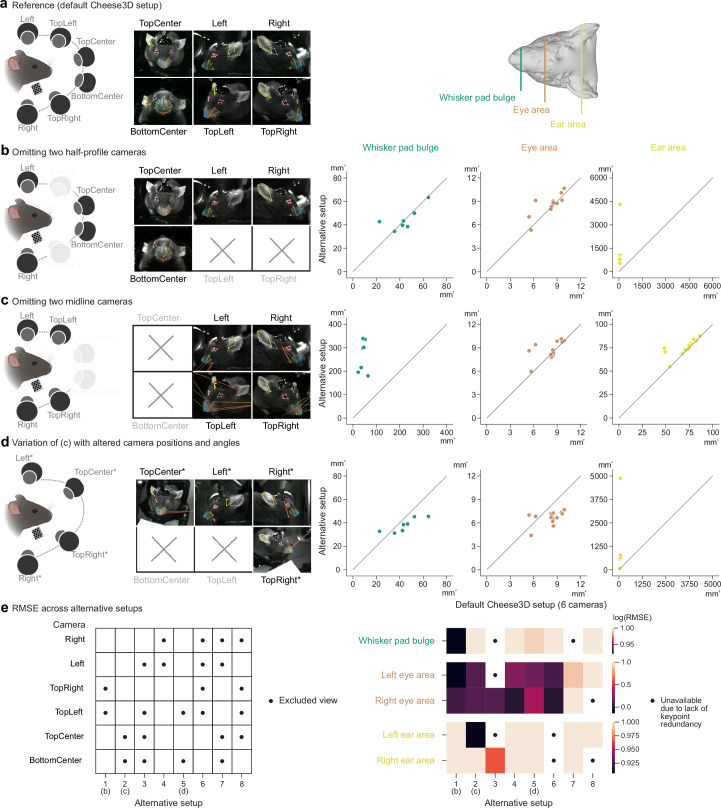
Utility and necessity of six cameras in capturing mouse face. (**a**) Default setup for Cheese3D with a calibrated array of six (three pairs of) cameras: hardware schematic (*left*) and 3D facial keypoints from the Cheese3D model projected onto example video frames (*right*), same as Fig. 1. Created in BioRender. CSHL, T. H. L. (2026) https://BioRender.com/6sr5jnd. (**b**) Omitting a pair of half-profile cameras results in altered keypoint inference and measurements of the most lateralized structures (Ear area RMSE: 1349.22 *μ*m^2^) but intact midline structures (Whisker pad bulge RMSE: 8.34 *μ*m^3^) and eye area (RMSE: 0.95 *μ*m^2^) compared to the default Cheese3D setup. Created in BioRender. CSHL, T. H. L. (2026) https://BioRender.com/6sr5jnd. (**c**)Omitting a pair of midline cameras results in distorted keypoint inference and measurements of midline structures (Whisker pad bulge RMSE: 231.92 *μ*m^3^) but intact lateralized structures (Eye area RMSE: 1.27 *μ*m^2^; Ear area RMSE: 9.22 *μ*m^2^) compared to the default Cheese3D setup (Fig. 1i). Created in BioRender. CSHL, T. H. L. (2026) https://BioRender.com/6sr5jnd. (**d**) A variation of setup of (**c**) with altered camera positions and angles does not rescue keypoint inference and measurements of the most lateralized structures (Ear area RMSE: 1311.45 *μ*m^2^) but has similarly intact midline structures (Whisker pad bulge RMSE: 9.89 *μ*m^3^) and eye area (RMSE: 1.90 *μ*m^2^). Created in BioRender. CSHL, T. H. L. (2026) https://BioRender.com/6sr5jnd. (**e**) Summary of measurement error across eight setups excluding certain combinations of cameras. *Left:* Cameras excluded in each setup. Setup 1 corresponds to (**b**), setup 2 corresponds to (**c**), setup 5 corresponds to (**d**). *Right:* Log-RMSE of setups (capped at $1=\log (10)$) grouped by facial feature. Facial features that could not be computed due to lack of keypoint redundancy are represented by a dot. Source data.

To validate the utility and necessity of having all six cameras, we omitted different pairs of cameras and measured changes in accuracy in corresponding facial regions (Extended Data Fig. 3). Omitting frontal cameras resulted in skewed measurements of midline facial features (for example, whisker pad bulge), whereas omitting elevated half-profile cameras resulted in errors of the most lateral features (for example, ears). Comparatively, two-camera setups generally failed to capture any features, except for some setups that captured lateral features (for example, eyes; Extended Data Fig. 3e). The six-camera array is also essential as it builds in redundancy, which ensures that measurements are still possible even when part of the face is obstructed in some views, as is often the case when the mouse paws (for example, during grooming) or experimental apparatuses (for example, to deliver food, drugs, or olfactory stimuli) come into close proximity to the face. Collectively, the synchronized and calibrated array of six cameras, combined with geometric facial features in 3D, reduces the tradeoff between compromising spatial versus temporal resolution in characterizing rodent facial movement.

### Reduction of tracking noise enables precise measurement of subtle and transient movements across facial regions

**Fig. 2:**
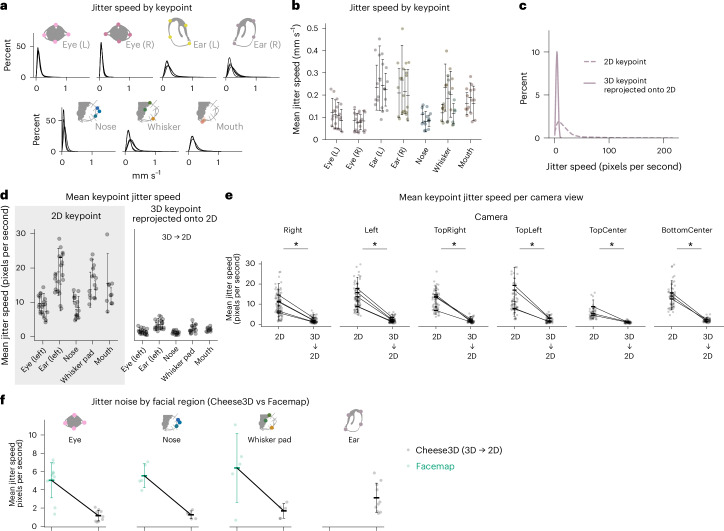
Reduction in keypoint tracking jitter due to 3D triangulation of data from six camera views. **a**, Distribution of keypoint-specific jitter (frame-to-frame speed) during a motionless period for an example mouse. Each subpanel indicates a different facial region, and each curve indicates a different keypoint. Created in BioRender; CSHL, T. H. L. https://BioRender.com/6sr5jnd (2026). **b**, Summary of keypoint-specific jitter across mice (*n* = 5). Each column group indicates a facial region, and each column indicates a keypoint. Grouped by facial regions (mean ± s.d.) for *n* = 5 mice: ear (left), 0.24 ± 0.10 mm s^−1^; ear (right), 0.22 ± 0.11 mm s^−1^; eye (left): 0.11 ± 0.06 mm s^−1^; eye (right), 0.08 ± 0.04 mm s^−1^; nose, 0.09 ± 0.04 mm s^−1^; whisker pad, 0.17 ± 0.09 mm s^−1^; mouth: 0.17 ± 0.06 mm s^−1^. **c**, Reduction of keypoint-specific jitter for an example keypoint from the left ear region in left camera view for an example mouse in 2D keypoints (before triangulation, dashed line) compared to 3D keypoints reprojected onto the 2D camera view plane (after triangulation, solid line). **d**, Changes in jitter for all keypoints from one example view (left camera) across mice (*n* = 5) between pre-triangulation (2D keypoints, shaded region) and post-triangulation (reprojected 2D keypoints). See Extended Data Fig. 4 for all views and summary statistics. **e**, Summary of keypoint-specific jitter across mice and all six camera views. See Extended Data Fig. 4 for summary statistics. All *P* < 0.05, one-sided Wilcoxon matched-pairs test, 2D keypoints > 3D keypoints reprojected onto 2D (‘*’ indicates significance). **f**, Jitter comparison between Cheese3D and Facemap across facial regions. Each point represents the mean jitter speed across all keypoints for the facial regions as well as camera views for a single mouse. Cheese3D’s 3D keypoints are reprojected onto 2D views to compare with Facemap. Note that Facemap’s output does not include the mouse’s ears. Measurements for lateralized facial features (eyes, ears) contain both left and right sides and thus have twice the number of data points compared to midline features (nose, whisker pad). See Extended Data Fig. 6 for the same data broken down by camera view. For Facemap: eye, 5.04 ± 1.92 pixels per second; nose, 5.53 ± 1.32 pixels per second; whisker pad, 6.39 ± 3.78 pixels per second; for Cheese3D: eye: 1.17 ± 0.59 pixels per second; nose, 1.26 ± 0.44 pixels per second; whisker pad, 1.69 ± 0.80 pixels per second; ear, 3.12 ± 1.61 pixels per second; *n* = 5 mice. *P* < 0.005, one-sided Wilcoxon matched-pairs test (Facemap > post-triangulation Cheese3D). Created in BioRender; CSHL, T. H. L. https://BioRender.com/6sr5jnd (2026). Source data.

**Extended Data Fig. 4:**
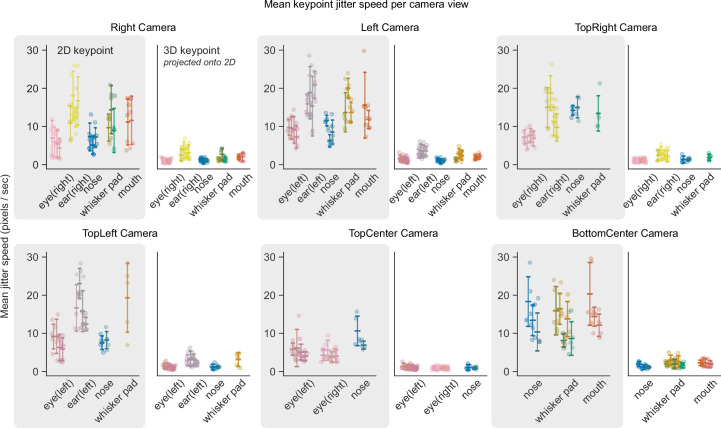
Keypoint jitter comparison between 2D and 3D. Summary of keypoint-specific jitter where each pair of panels represents a different camera view, and each pair shows the jitter pre- (shaded) and post-triangulation (Mean jitter speed, pre-triangulation. Ear (left): $17.37\pm 5.86\,{\rm{px}}/\sec$, Ear (right): $14.12\pm 5.26\,{\rm{px}}/\sec$, Eye (left): $7.29\pm 3.39\,{\rm{px}}/\sec$, Eye (right): $5.67\pm 2.67\,{\rm{px}}/\sec$, Nose: $10.05\pm 4.68\,{\rm{px}}/\sec$, Whisker pad: $13.55\pm 5.86\,{\rm{px}}/\sec$, Mouth: $13.86\pm 6.12\,{\rm{px}}/\sec$; Mean jitter speed, post-triangulation. Ear (left): $3.16\pm 1.37\,{\rm{px}}/\sec$, Ear (right): $2.85\pm 1.50\,{\rm{px}}/\sec$, Eye (left): $1.29\pm 0.67\,{\rm{px}}/\sec$, Eye (right): $1.02\pm 0.50\,{\rm{px}}/\sec$, Nose: $1.23\pm 0.55\,{\rm{px}}/\sec$, Whisker pad: $2.21\pm 1.15\,{\rm{px}}/\sec$, Mouth: $2.10\pm 0.74\,{\rm{px}}/\sec$; *n* = 5 mice. All *p* < 0.0001, one-sided Wilcoxon matched-pairs test (pre-triangulation > post-triangulation)). Source data.

**Extended Data Fig. 5:**
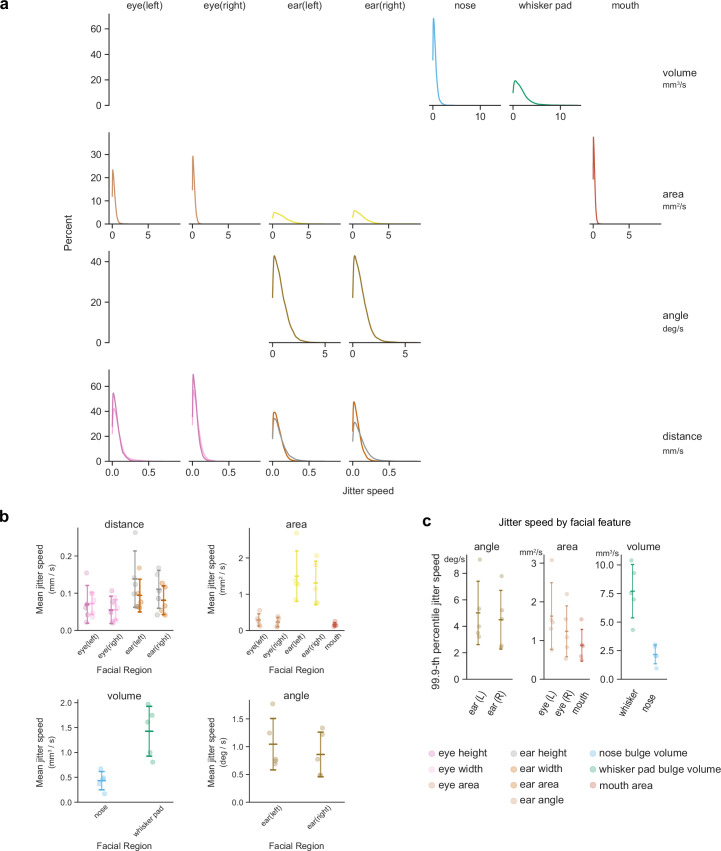
Tracking jitter by 3D facial feature. (**a**) Distribution of facial feature-specific jitter (frame-to-frame speed of keypoints) relative to the mean jitter during a motionless period for an example mouse. Each column indicates a different facial region, and each row indicates a different type of measurement. (**b**)Summary of facial feature-specific jitter where each panel represents a different type of measurement. (**c**)Summary of 99.9-th percentile jitter speed used in Fig. 3a,b. Mean ± std; Ear angle (left): $5.01\pm 2.3{9}^{\circ }/\sec$; Ear angle (right): $4.50\pm 2.2{2}^{\circ }/\sec$; Eye area (left): $1.63\pm 0.86\,{{\rm{mm}}}^{2}/\sec$; Eye area (right): $1.24\pm 0.66\,{{\rm{mm}}}^{2}/\sec$; Mouth area: $0.88\pm 0.41\,{{\rm{mm}}}^{2}/\sec$; Whisker pad bulge: $7.71\pm 2.33\,{{\rm{mm}}}^{3}/\sec$; Nose bulge: $2.18\pm 0.83\,{{\rm{mm}}}^{3}/\sec$; *n* = 5 mice. Source data.

**Extended Data Fig. 6:**
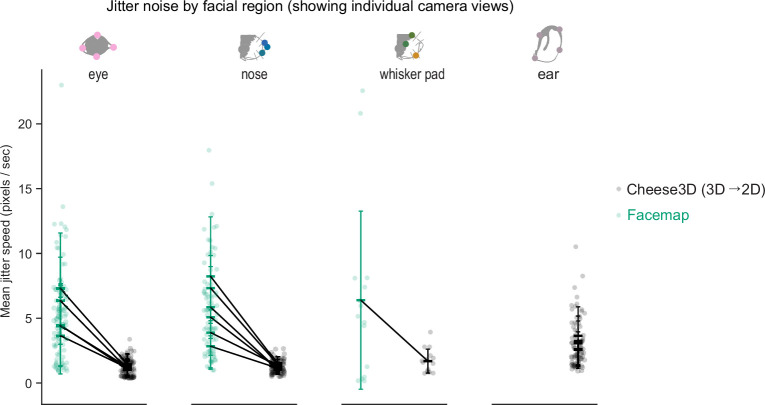
Reduction in keypoint tracking jitter from Facemap to Cheese3D by facial region. Comparison of jitter between Facemap’s output and Cheese3D’s output. Each datapoint represents the mean jitter speed of a single mouse for a particular facial region and the camera views where the region is visible from. (Mean ± std jitter speed, Facemap. Eye (Left view): $7.29\pm 4.29\,{\rm{px}}/\sec$, Eye (Right view): $4.46\pm 3.19\,{\rm{px}}/\sec$, Eye (TopRight view): $3.65\pm 2.95\,{\rm{px}}/\sec$, Eye (TopCenter): $6.35\pm 3.36\,{\rm{px}}/\sec$, Eye (TopLeft): $4.42\pm 3.09\,{\rm{px}}/\sec$, Nose (Left view): $5.08\pm 3.90\,{\rm{px}}/\sec$, Nose (Right view): $2.83\pm 1.77\,{\rm{px}}/\sec$, Nose (TopCenter view): $5.85\pm 2.41\,{\rm{px}}/\sec$, Nose (TopRight view): $8.23\pm 4.59\,{\rm{px}}/\sec$, Nose (TopLeft view): $3.89\pm 1.75\,{\rm{px}}/\sec$, Nose (BottomCenter view): $7.32\pm 2.51\,{\rm{px}}/\sec$, Whisker Pad (Right view) $6.39\pm 6.87\,{\rm{px}}/\sec$; Mean jitter speed, Cheese3D. Eye (Left view): $1.41\pm 0.80\,{\rm{px}}/\sec$, Eye (Right view): $1.07\pm 0.57\,{\rm{px}}/\sec$, Eye (TopRight view): $1.15\pm 0.60\,{\rm{px}}/\sec$, Eye (TopCenter view): $1.07\pm 0.59\,{\rm{px}}/\sec$, Eye (TopLeft view): $1.22\pm 0.61\,{\rm{px}}/\sec$, Nose (Left view): $1.10\pm 0.42\,{\rm{px}}/\sec$, Nose (Right view): $1.15\pm 0.42\,{\rm{px}}/\sec$, Nose (TopCenter view): $1.05\pm 0.34\,{\rm{px}}/\sec$, Nose (TopRight view): $1.48\pm 0.55\,{\rm{px}}/\sec$, Nose (TopLeft view): $1.34\pm 0.48\,{\rm{px}}/\sec$, Nose (BottomCenter view): $1.43\pm 0.58\,{\rm{px}}/\sec$, Whisker Pad (Right view): $1.69\pm 0.92\,{\rm{px}}/\sec$, Ear (Left view): $3.64\pm 2.24\,{\rm{px}}/\sec$, Ear (Right view): $3.25\pm 1.91\,{\rm{px}}/\sec$, Ear (TopRight view): $2.55\pm 1.39\,{\rm{px}}/\sec$, Ear (TopLeft view): $3.04\pm 1.74\,{\rm{px}}/\sec$; *n* = 5 mice. All *p* < 0.005, one-sided Wilcoxon matched-pairs test (pre-triangulation > post-triangulation)). Created in BioRender. CSHL, T. H. L. (2026) https://BioRender.com/6sr5jnd. Source data.

To assess the sensitivity of Cheese3D to detect and measure small, localized facial movements, we sought to explicitly quantify keypoint jitter in our setup in a control experiment using motionless periods during anesthesia. Keypoint jitter is a known issue whereby local fluctuations in keypoint tracking are unrelated to genuine movement^27^. This can happen as a result of image noise, inadequate lighting, low contrast or texture, label noise in the training data or keypoint-specific uncertainty in the model. Across the facial keypoints selected, human labelers use not only texture, but also color and shape to determine the location of keypoints. Convolutional neural networks often focus on texture to solve object recognition tasks^28^; therefore, it should be expected that certain keypoints that rely primarily on texture for their location are learned more confidently than others. Keypoint jitters are often mitigated using low-pass filters, but this can attenuate dynamics and reduce the temporal resolution of detection^27^. A critical benefit of 3D multi-view calibration compared to single 2D uncalibrated views is that view redundancy reduces the amplitude of keypoint jitter, allowing us to detect more subtle, fast movements. Studying motionless periods, we detected jitters of 3D keypoints without any filtering (Fig. 2a,b) and measured the reduction in jitter between 2D keypoints and 3D keypoints projected onto 2D views (Fig. 2c–e; see Extended Data Fig. 4 for more details). Additionally, we processed the same video data using an existing 2D mouse face pose tracking tool: Facemap^3^ (Fig. 2f and Extended Data Fig. 6). In all comparisons, we observed a reduction in keypoint jitter after 3D triangulation. We further examined the effect of keypoint jitter on geometric features, which informs the mouse-specific threshold between keypoint tracking noise and bona fide movements that Cheese3D can detect (Extended Data Fig. 5).

### Uncovering underlying physiology from external facial movements

**Fig. 3:**
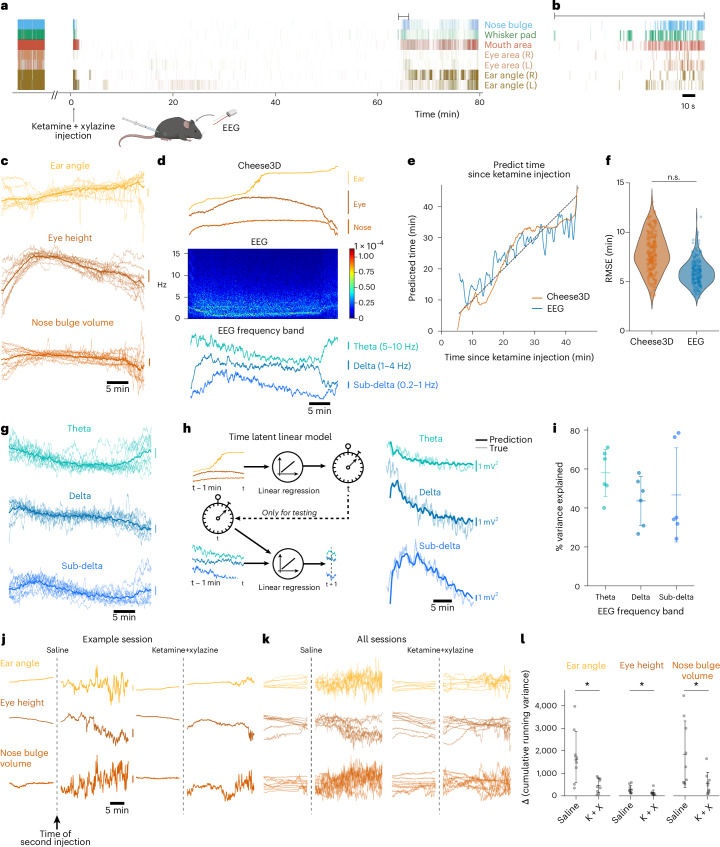
Distinct facial patterns track time during induction and recovery from ketamine-induced anesthesia. **a**, Example facial movement raster plot during anesthesia with concurrent EEG recording (each vertical line corresponds to movement above the 99.9th percentile jitter threshold as shown in Extended Data Fig. 5c for a given 10 ms time window). Created in BioRender; CSHL, T. H. L. https://BioRender.com/6sr5jnd (2026). **b**, Zoom in of movement raster plot from **a** to show the early moments of movement recovery following anesthesia. **c**, Overlaid *z*-scored ear angle, eye height and nose bulge volume traces from 12 sessions (*n* = 3 mice; four sessions per mouse; vertical scale bar indicates 1 s.d.). **d**, Simultaneously recorded Cheese3D features (top, showing moving average over a 10 s window; vertical scale bars: ear, 2^∘^; eye, 0.1 mm; nose, 0.5 mm^3^), EEG spectrogram (middle, 5 s FFT window) and power of EEG frequency bands (bottom: showing sub-delta, 0.2–1 Hz; delta, 1–4 Hz; and theta, 5–10 Hz bands; vertical scale bars indicate 1 s.d.) for an example session. **e**, Output from a quadratic model fit across mice predicting time since injection using the initial and current Cheese3D or EEG feature values relative to the dotted identity line. **f**, RMSE of time prediction in which each dot represents the mean test error for one particular model trained on either Cheese3D or EEG features. Mean ± s.d.: 7.91 ± 1.95 min for Cheese3D, 5.90 ± 1.27 min for EEG; four sessions per mouse, *n* = 3 mice. *P* = 0.068 (n.s., not significant), paired two-sided *t*-test with *k*-fold cross-validation correction as per previous work^56,57^. **g**, Overlaid *z*-scored theta, delta and sub-delta frequency band power traces from 12 sessions (same as in **c**; vertical scale bars indicate 1 s.d.). **h**, Prediction of EEG frequency band power from facial features. Left, sketch of the time-latent linear model used to predict EEG frequency band power signals from ear angle, eye height and nose bulge volume. Two separate linear regression fits are trained to either predict time elapsed since injection using facial features in the past 1 min, or predict current EEG frequency band power signal using past EEG frequency band power signals and time elapsed since injection. Right, example predictions on a held-out recording of each EEG frequency band. **i**, Variance explained in each EEG frequency band power using a time-latent linear model of ear angle, eye height and nose bulge volume during held-out recordings. Mean ± s.d.: theta, 57.99 ± 11.99%; delta, 43.61 ± 12.49%; sub-delta, 46.69 ± 24.14%; *n* = 6 mice, 29 total sessions. **j**, Differences in *z*-scored facial features in an example mouse comparing ketamine + xylazine re-dosing versus saline control (vertical scale bar indicates 1 s.d.). Data from ±100 s around the second injection (dashed line) are excluded since wireless communication with the recording device is unreliable during these periods. **k**, Overlaid *z*-scored ear angle, eye height and nose bulge volume traces (*n* = 4 mice, 18 total sessions; vertical scale bar indicates 1 s.d.) separated by the second injection type (saline vs ketamine + xylazine). **l**, Differences between the cumulative running variance of each facial feature 15 min before or after the second injection, averaged over a 5 min window. Values represent means ± s.d. Ear angle: saline, 1,731.92 ± 1,128.28^∘2^; ear angle: K + X, 470.58 ± 317.94^∘2^; eye height: saline, 294.93 ± 175.21 mm^2^; eye height: K + X, 133.73 ± 127.73 mm^2^; nose bulge volume: saline, 1,846.91 ± 1,479.53 mm^6^; nose bulge volume: K + X, 559.98 ± 486.10 mm^6^; *n* = 4 mice, 18 total sessions. *P* < 0.01, two-sided Mann–Whitney test with subject group permutation correction (‘*’ indicates significance). Source data.

As a proof-of-principle test that Cheese3D is able to capture subtle and rapid facial movements with physiological significance, we designed experiments to monitor mice emerging from ketamine-induced general anesthesia. Small localized facial movements, including whisker deflections, appear during anesthesia and signal early stages of recovery^29^. This poses unique challenges to sensitively track subtle movements with coverage of the entire face compared to other overt body movements, such as locomotion and reaching, where limbs and appendages undergo large translations and rotations relative to their size. Minimizing tracking jitter, as described in the previous section, is essential to track such movements. We applied the thresholds defined by the jitter analysis (see Extended Data Fig. 5c) to facial movements recorded by Cheese3D during anesthesia. A detailed examination of facial movement speed during anesthesia revealed temporal patterns across different facial regions. Motions within different facial regions are visualized in the movement raster plot, where each vertical line represents displacement above a jitter threshold in the corresponding video frame (we selected one representative geometric feature for each of the seven facial regions) (Fig. 3a,b). These data demonstrate that Cheese3D can be used to detect small movements associated with anesthesia and recovery in mice.

We examined whether Cheese3D can be used to study facial movements associated with physiological processes that are not otherwise externally visible. Visualizing facial features over the entire anesthetic period from induction to recovery revealed gradual changes in facial motion (for example, ears and eyes) that are stereotyped across sessions and across mice (Fig. 3c and Supplementary Video 4). This suggests that certain facial features can be used as a ‘stopwatch’ across mice to track time since anesthesia induction. To test this hypothesis, we fit a single model across all mice to predict the time elapsed since anesthesia induction (intraperitoneal injection of ketamine and xylazine) using only the initial and current value of filtered (10 s moving average window) facial features (Fig. 3e,f). As a comparison, we recorded temporally synchronized electroencephalography (EEG) and fit the same model using power from EEG frequency bands (Fig. 3d,g), which serves as an indicator of brain state during anesthesia^30^. We observe that Cheese3D facial features allow us to predict time since anesthesia induction with similar performance to using EEG features (Fig. 3f).

**Extended Data Fig. 7:**
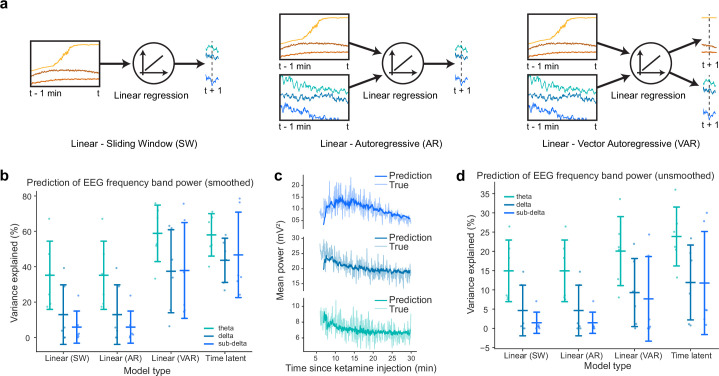
Predicting EEG frequency band power from facial features using various linear models. (**a**)Sketches of alternative models studied in addition to Fig. 3h. (**b**)Variance explained in each EEG frequency band power (smoothed using ≈ 200 ms moving average filter) using all linear models in (**a**) of ear angle, eye height, and nose bulge volume during held-out recordings. Mean ± std; linear (SW), theta: 35.16 ± 19.28%; linear (SW), delta: 12.93 ± 16.83%; linear (SW), sub-delta: 5.86 ± 9.07%; linear (AR), theta: 35.16 ± 19.28%; linear (AR), delta: 12.93 ± 16.83%; linear (AR), sub-delta: 5.86 ± 9.07%; linear (VAR), theta: 58.85 ± 15.97%; linear (VAR), delta: 37.44 ± 23.47%; linear (VAR), sub-delta: 36.20 ± 28.97%; *n* = 6 mice, 29 total sessions. (**c**)Example unsmoothed predictions on a held-out recording of each EEG frequency band powers from ear angle, eye height, and nose bulge volume using a linear model with time as a latent variable. (**d**)Variance explained in each EEG frequency band power (unsmoothed) using all linear models in (**a**) of ear angle, eye height, and nose bulge volume during held-out recordings; linear (SW), theta: 14.94 ± 8.00%; linear (SW), delta: 4.64 ± 6.57%; linear (SW), sub-delta: 1.46 ± 2.76%; linear (AR), theta: 14.94 ± 8.00%; linear (AR), delta: 4.64 ± 6.57%; linear (AR), sub-delta: 1.46 ± 2.76%; linear (VAR), theta: 20.07 ± 8.96%; linear (VAR), delta: 9.30 ± 8.84%; linear (VAR), sub-delta: 7.67 ± 10.99%; time-latent (AR), theta: 23.85 ± 7.65%; time-latent (AR), delta: 11.92 ± 9.72%; time-latent (AR), sub-delta: 11.77 ± 13.38%; *n* = 6 mice, 29 total sessions. Source data.

To further quantify how well facial features track anesthetic state, we sought to predict moment-to-moment changes in each EEG frequency band power using the three facial features in Fig. 3c. Building on our findings in Fig. 3e,f, we trained a linear model from a sliding window history of facial features to the current EEG power using time since injection as a latent factor (model illustrated in Fig. 3h; alternative models and results in Extended Data Fig. 7). We find that we can explain 57.99 ± 11.99%, 43.61 ± 12.49% and 46.69 ± 24.14% (mean ±s.d.) of the variance in the theta, delta and sub-delta frequency bands, respectively (Fig. 3h,i; *n* = 6 mice, 29 total sessions).

To determine whether facial features can be used as a robust marker of anesthetic state, we performed an experiment in which mice were anesthetized as in Fig. 3a, but then injected a second time after 30 min with either half of the original dosage of ketamine + xylazine or a control saline solution. We observed clear differences in the ear angle, eye height and nose bulge volume after the second injection between the control and re-dosed groups (Fig. 3j,k). Measuring the mean cumulative running variance of these features before and after re-dosing, we observed significant differences between the control and re-dosed groups, indicative of anesthetic state (Fig. 3l). In short, facial features from Cheese3D track small, moment-to-moment changes in brain state in anesthetized mice, serving as a potential noninvasive measure of anesthetic depth.

We tested Cheese3D with facial movements that are vigorous in amplitude: chewing in rodents is difficult to characterize externally, given that the teeth, along with food that has entered the mouth, cannot be seen. However, being able to track and measure chewing is essential to studies of nutrient absorption and efficient digestion^31^ as well as jaw proprioception^32^. Although licking has been tracked noninvasively^18,33,34^, existing techniques to characterize chewing rely on invasive methods such as electromyography to infer what is happening inside the mouth^35^. We hypothesized that Cheese3D would enable more direct assessment of chewing dynamics from careful examination of external facial movements during food consumption.

**Fig. 4:**
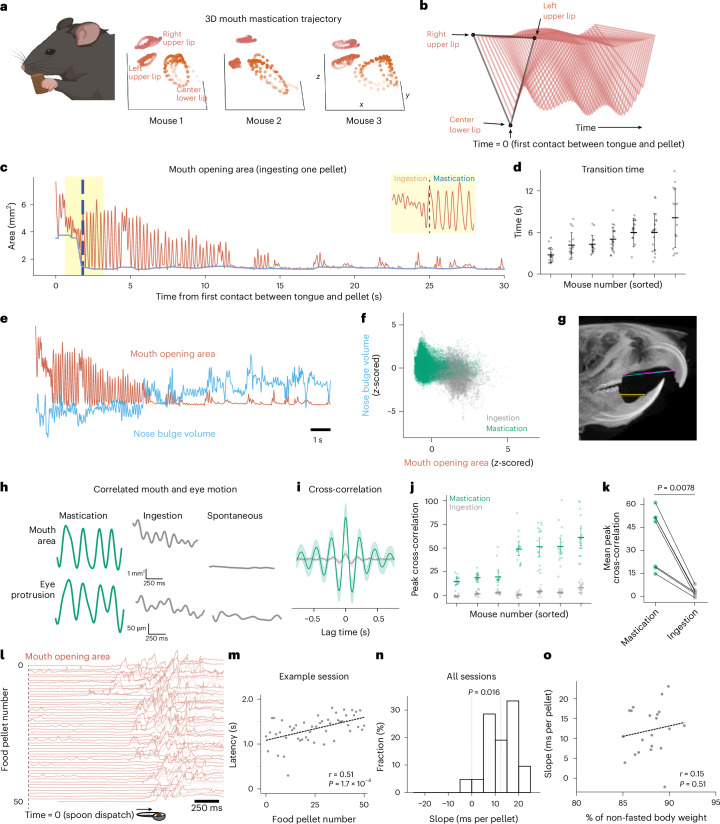
Chewing kinematics in mouth and surrounding facial areas. **a**, 3D trajectory of three mouth keypoints for 1 s of chewing motion for three example mice. Created in BioRender; CSHL, T. H. L. https://BioRender.com/6sr5jnd (2026). **b**, Time evolution of the mouth opening triangle formed by the three mouth keypoints in **a** for 0.5 s from the moment the food pellet comes into contact with the tongue in an example mouse. **c**, Area of the mouth opening triangle over time during consumption of one pellet for an example mouse, with a zoomed-in view around the transition time shown in the inset. **d**, Summary of ingestion to mastication transition times. Each column is one mouse; each dot is one food pellet. Mean ± s.d., 5.20 ± 1.71 s, ranging from 2.77 s to 8.12 s; *n* = 7 mice. **e**, Visualizing mouth opening area concurrent with nose bulge volume (*z*-scored per feature) while an example mouse consumes a single pellet (same pellet as shown in **c**). **f**, Same data as in **e**; each dot represents a time point before or after the transition time. **g**, MicroCT image of the mouse with diastema, the gap between incisors (for ingestion) and molars (for mastication), labeled in color lines. **h**, Example time segments of mouth area with eye protrusion during putative mastication and ingestion, and during spontaneous movement outside of chewing. **i**, Cross-correlation between mouth area and eye protrusion for one example session for putative mastication and ingestion phases. **j**, Summary of peak cross-correlation (computed as shown in **i**) across pellets, where each column is one mouse. Mean ± s.d., 38.05 ± 19.45 for mastication, 2.80 ± 2.81 for ingestion; *n* = 7 mice. **k**, Summary of mean peak cross-correlation (computed as shown in **h**) across pellets; each point is one mouse (*n* = 7 mice). *P* < 0.001, one-sided Wilcoxon matched-pairs test (mastication mean value > ingestion mean value). **l**, Mouth opening area over 50 pellets for one example session. Time at zero indicates when the spoon is dispatched (dashed line). **m**, Time to first mouth opening over threshold by pellet number for the session in **l** and the corresponding linear fit. Pearson correlation coefficient, *r* = 0.51, *P* = 1.7 × 10^−4^, unpaired two-sided *t*-test. **n**, Distribution of slopes from linear fits as in **m** across all sessions (*n* = 7 mice, 21 total sessions). Dashed line at zero indicates the null hypothesis (that is, median slope = 0), and dashed line at 12.39 indicates the median slope. *P* = 0.016, two-sided Wilcoxon unpaired test. **o**, The slope of each linear fit plotted against the body weight of each mouse relative to their non-fasted weight. Pearson correlation coefficient, *r* = 0.15, *P* = 0.51, unpaired two-sided *t*-test. Source data.

Using the same Cheese3D multi-camera array and 17 geometric facial feature identification system with no modifications, we recorded mice as they ate crunchy food (3 mm diameter precision pellets) and visualized the 3D trajectory of mouth keypoints (upper lip corners and lower lip, forming a triangle in 3D space; Fig. 4a,b). Plotting the area of this triangle (that is, mouth opening) over time revealed two distinct modes of eating with either elevated or reduced lower signal envelope, corresponding, respectively, to a food pellet obstructing the mouth opening or the mouth shut (Fig. 4c). The transition between the two modes is abrupt and reliably identifiable across all mice (Fig. 4d and Supplementary Video 5). The clear separation is also evident in movements within the facial area close to the back of the mouth (Fig. 4e,f). This finding is consistent with the unique tooth anatomy of rodents, in which a distinct gap, termed diastema, separates the incisors (for ingestion) from the molars (for mastication), as labeled in microCT images (Fig. 4g). Whole-face movement analysis also revealed temporally correlated eye protrusion with chewing during mastication but not ingestion or other spontaneous facial movement for every mouse examined (Fig. 4h–k and Supplementary Video 6). This could potentially be attributed to the anatomy of the rodent muscles of mastication, given that they wrap around the base of the eye socket^26^. The phenomenon has been frequently observed and named ‘eye boggling’ in the pet rodent community and only recently described in scientific literature^36^. Our data indicate that Cheese3D detects facial movements during rodent food consumption consistent with known characteristics of food placement, tooth anatomy and muscle engagement.

**Extended Data Fig. 9:**
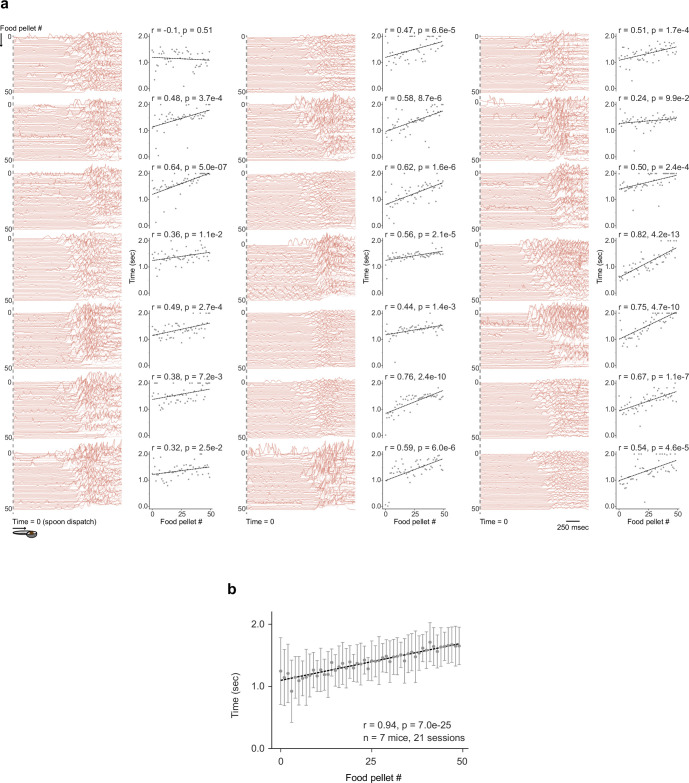
Mouth opening area across 50 pellets for all mice and sessions. (**a**)*Left per column:* Mouth opening area over 50 pellets across all mice and sessions (*n* = 7 mice, 21 total sessions). Time = 0 indicates when the spoon is dispatched (dashed line). *Right per column:* Time to first mouth opening above threshold by pellet number for all mice and sessions and the corresponding linear fits. (**b**)Time to first mouth opening over threshold averaged across all sessions by pellet number and the linear fit. (n = 7 mice, 21 total sessions). Source data.

Additionally, to determine whether Cheese3D can detect changes in fine facial kinematics over time during food consumption, which has been shown to correlate with changes in brain activity and homeostatic states^37–44^, we systematically tracked the facial movements of food-restricted mice as they were offered 50 pellets per session, with an inter-pellet interval of 30 s (*n* = 7 mice, 21 total sessions). As in the previous experiment, a spoon dispatched the pellets, taking approximately 1.8 s to be within reach of the mouse’s mouth. We used the time after spoon dispatch, where the mouth area crosses above 3 mm^2^ to measure initiation of food ingestion (Fig. 4l). As more pellets were delivered, we observed a significant increase in latency in ingestion-related mouth opening after spoon dispatch (Fig. 4l,m), which may result from mice being more satiated. This increase in latency was consistent across mice (Fig. 4n and Extended Data Fig. 9). Still, the rate of change in latency of mouth opening does not significantly correlate with the strength of food restriction in the range we tested (85–91% of non-fasted body weight; Fig. 4o). Overall, analysis of the Cheese3D facial features reveals subtleties about consummatory behavior.

### Linking facial movement to motor control machineries using synchronized Cheese3D with electrophysiology

**Fig. 5:**
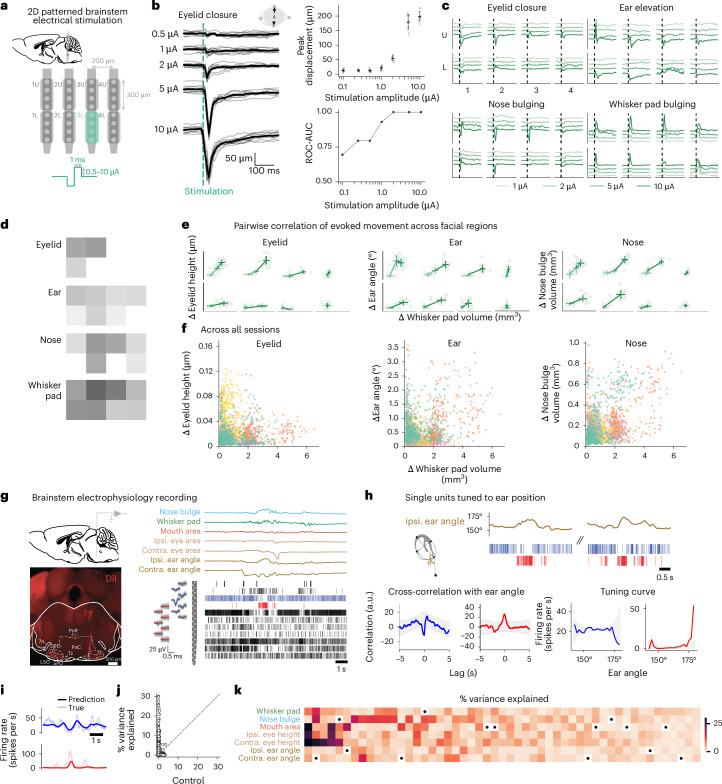
Synchronized Cheese3D with electrophysiology relates motor control activity to subtle facial movements. **a**, Schematic showing the brainstem targeted by a four-shank silicon probe (image of brain taken from a previous publication^58^). Electrodes are divided into a two-by-four grid to allow for patterned stimulation of the targeted area. Created in BioRender; CSHL, T. H. L. https://BioRender.com/6sr5jnd (2026). **b**, Example traces of ipsilateral eyelid closing triggered by bulk electrical stimulation across varying current amplitudes (*n* = 10 stimulations per amplitude, left) with peak absolute deflection measured as a function of stimulation amplitude (top right). Mean ± s.d. by amplitude: 0.1 μA, 12.32 ± 5.10 μm; 0.25 μA, 12.80 ± 3.70 μm; 0.5 μA, 12.07 ± 4.52 μm; 1.0 μA, 20.99 ± 7.06 μm; 2.0 μA, 54.55 ± 8.69 μm; 5.0 μA, 179.27 ± 26.13 μm; 10 μA, 199.01 ± 16.84 μm. Area under the receiver operating characteristic curve (ROC-AUC) when distinguishing pre-stimulation responses from post-stimulation responses for varying stimulation amplitudes (bottom right). Created in BioRender; CSHL, T. H. L. https://BioRender.com/6sr5jnd (2026). **c**, Responses across facial features to spatial-specific focal stimulation at varying amplitudes. Each trace indicates the mean response per amplitude (*n* = 15–30 stimulations per amplitude and location). **d**, Peak absolute deflection of responses in **c** at 10 μA showing spatially localized movement for different facial features. **e**, Pairwise relationship of peak absolute deflections of responses in **c** relative to whisker pad volume. See Supplementary Table 4 for summary statistics. **f**, Pairwise relationship of peak absolute deflections of responses as in **e** but colored by experimental sessions (eight sessions from *n* = 4 mice). **g**, Schematics of in vivo electrophysiological recordings (top left, image of brain taken from a previous publication^58^) with silicon probe track revealed by DiI in the brainstem PnC (bottom left). Example recording of facial movements and single-unit activity in the brainstem of awake mice (*n* = 4 mice, nine sessions, 77 units). PnC: pontine reticular nucleus caudalis. PnR: pontine raphe nucleus. DPO: dorsal periolivary region. LSO: lateral superior olive. SPO: superior paraolivary nucleus. Tz: nucleus of the trapezoid body. 7n: facial nerve. Created in BioRender; CSHL, T. H. L. https://BioRender.com/6sr5jnd (2026). **h**, Two example single units with anti-correlated activities that are both tuned to ear angle (subset of units in **g**). Neural activities (top), cross-correlation with ipsilateral ear angle (bottom left) and tuning curves (right) for the two example units. The lag of peak correlation is −20 ms and −80 ms, respectively. Gray shading in cross-correlation: 99.9% confidence interval (CI) of shuffled data. Gray shading in tuning curve: 95% CI of shuffled data. a.u., arbitrary units. Created in BioRender; CSHL, T. H. L. https://BioRender.com/6sr5jnd (2026). **i**, Predicted instantaneous firing rate of example units in **h** from ipsilateral ear angle (smoothed with a 200 ms moving average window). **j**, Variance explained of the smoothed firing rate of single units predicted from nose bulge volume, whisker pad volume, mouth area, ipsilateral and contralateral eye height or ipsilateral and contralateral ear angle (*n* = 4 mice, nine sessions, 77 units). Controls are the mean of 1,000 models fit on shuffled spike times using 50 ms chunks. Each dot represents a specific facial feature and unit pair. **k**, Variance explained for significant results in **j** organized by facial feature and unit pairs (*P* ≥ 0.05 by one-sided permutation test on controls in **j** are marked with a dot; true fit variance explained > control fit variance explained). Only units for which the maximum variance explained by any facial feature is more than 2% are shown. Source data.

Facial musculature is controlled by motor neurons originating from the brainstem motor network. As such, relating facial movement to facial motor control machineries is a key first step to delineating the mechanisms linking brain-wide activity to facial behavior. Thus far, we have demonstrated that Cheese3D is accurate and precise enough to reveal internal physiological processes such as anesthetic depth and food ingestion phases. Yet facial movements are unique in that large internal differences can manifest as subtle changes between two nearly identical movements. To quantify whether Cheese3D has the precision to detect such distinctions, we designed an experiment to induce small, localized movements in anesthetized mice by focal electrical stimulation of the brainstem motor control network using a four-shank silicon probe. By varying the current amplitude and location of stimulation, we aimed to create minute differences in facial responses, engaging the building blocks of facial movement across different regions (Fig. 5a).

**Extended Data Fig. 10:**
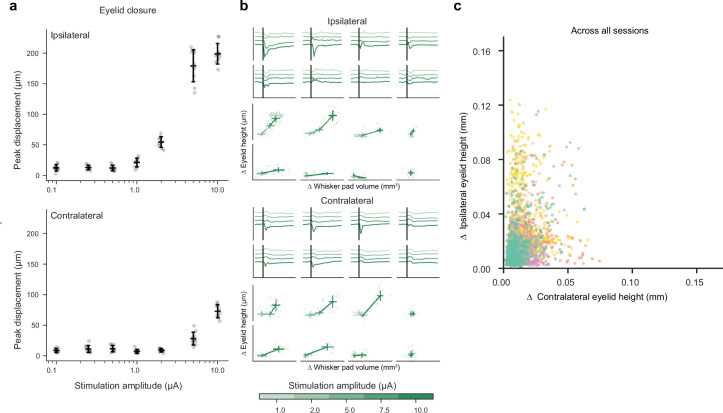
Ipsilateral vs. contralateral asymmetry of electrically stimulated facial movement. (**a**)Peak absolute deflection of ipsilateral eyelid closure (top) and contralateral eyelid closure (bottom) measured as a function of stimulation current amplitude. (**b**) Top half, from top to bottom: Ipsilateral eyelid closure responses to spatial-specific focal stimulation at varying amplitude; each trace indicates the mean response per amplitude (*n* = 15-30 stimulations per amplitude and location). Pairwise responses of ipsilateral eyelid closure vs. whisker pad volume responses. Bottom half: same as top but for contralateral eyelid responses. (**c**)Pairwise relationship of peak absolute deflections of responses for ipsilateral vs. contralateral eyelids, but colored by experimental sessions (8 sessions from *n* = 4 mice). Source data.

We next wanted to determine the smallest facial movement that Cheese3D could reliably detect. First, to ensure that Cheese3D can detect controlled differences in facial movement, we bulk-stimulated the entire region targeted by our probe at varying current amplitudes. We observed deflections in various facial features, such as the distance between eyelids (called ‘eye height’ in our tool) (Fig. 5b). Notably, we detected changes in eye height as small as 2.66 μm that increased with stimulation current, suggesting that Cheese3D can measure subtle differences in facial movement caused by neuronal activity. Our ability to distinguish such movements from pre-stimulation null responses improves with stimulation current amplitude (Fig. 5b, bottom right). Next, we applied focal stimulation at varying current amplitudes in an effort to target the building blocks of facial movement in the brainstem. Although we continued to observe current amplitude-dependent changes, we also observed spatially localized movement responses for different facial features (Fig. 5c,d). Cheese3D facial features include both highly local features (for example, eye height) that relate to localized musculature (for example, eyelid closure)^45^ and composite features (for example, whisker pad volume) that cover a larger region of the face and are affected by the activity of multiple muscles. For these composite features, we see movements caused by stimulation for all locations, but the modulation of responses is location-specific (Fig. 5c,d). This highlights the ability of composite features to quantify complex whole-face motion. For example, when we examined the pairwise movement responses of various features against the whisker pad volume, we observed that locations with strong eye movement and whisker pad movement responses group into distinct current amplitude-dependent clusters (Fig. 5e). On the other hand, for regions with little eye movement, clusters do not form even though there are measurable deflections in the whisker pad volume. Across experimental sessions, we observe solely local feature responses as well as composite facial responses (Fig. 5f). Although local features describe the movement of a single facial region, composite features summarize how these parts work in synchrony across the face. In addition to local versus composite responses, we also observed asymmetry across the ipsilateral and contralateral sides of the face (Extended Data Fig. 10). These complex motions indirectly reflect underlying biological mechanisms for coordination; however, as stimulation-evoked motion has demonstrated, Cheese3D has the precision to be one piece of a comprehensive quantification of the building blocks of facial movement.

Electrophysiological recordings in awake mice synchronized with Cheese3D further demonstrated correlated brainstem neural activity with specific facial features during spontaneous movements (Fig. 5g). Measuring facial motion in calibrated 3D world coordinates enables detecting expressive facial features with complex movements in 3D space, such as elevation and abduction of the pinna (3D ‘ear angle’ in Cheese3D output). Indeed, we discovered single units in the pontine reticular nucleus caudalis tuned to ear movement, with firing rates adjusting to direction-specific ear movements (Fig. 5g,h, in blue and red; *P* < 0.001), as suggested by prior work^46,47^. Furthermore, we found that Poisson generalized linear models (GLMs) that take a single Cheese3D facial feature as input predict the neural activity of individual units recorded in the brainstem (Fig. 5i,k). The predictability of neural activity from facial features reflects the moment-to-moment precision of Cheese3D (Fig. 5i). From 36 units recorded across six sessions, we found that for all units, neural activity is predicted by single or multiple facial features, explaining up to 30.05% of the variance per unit from individual features (Fig. 5j,k). Notably, some units are predictable by several facial features, potentially indicating coordinated movement by individual muscles. Our estimates of explained variance included neural variability that may be unrelated to facial expression, such as internal co-fluctuations^48^, leading to underestimates of the stimulus-related explained variance. Thus, our results reflect a lower bound on the efficacy of Cheese3D as a temporally precise measure of brainstem activity.

Taken together, these results show that Cheese3D has the precision and accuracy to relate the activity of neurons in the brainstem to facial movements. We demonstrated this ability by both inducing movements through directed stimulation and relating recorded neural activity to spontaneous motion. In particular, we demonstrated the ability to measure small differences in nearly identical motions, illustrating the ability of Cheese3D to capture the subtleties of facial movements. Thus, Cheese3D is a powerful tool for uncovering the mechanism underpinning facial motion.

## Discussion

The goal of Cheese3D is to provide an interpretable framework for using mouse face-wide movement to discover underlying physiological functions across a wide range of applications. Recognizing the unique and underexplored potential to use whole-face dynamics as a noninvasive readout of moment-to-moment changes of body and brain states in mice, we crafted Cheese3D as a specialized high-resolution tool to study mouse facial movements, compared to and built upon emerging animal behavioral tracking methods aimed to generalize across body parts and species^21–23,49–54^. We package our pipeline as an easy-to-use tool with an interactive visualizer and demonstrate its ability to work across experimental setups. Moreover, in contrast to existing methods that focus on static facial images, motion of a subset of facial features or aggregates of orofacial behavior optimized to predict cortical neural activities^3,6^, Cheese3D is specifically designed to capture and represent whole-face movement while maintaining spatial and physical interpretability. The multi-camera array setup facilitates reliable, markerless identification of facial keypoints in 3D space and counteracts the occlusion and distortion found in single-camera setups (for example, during feeding, when paws hide the mouth). It also reduces keypoint jitter compared to 2D methods, enables detection of subtle movements (for example, under anesthesia) and allows accurate capture of inherently 3D motion (for example, mouth opening during chewing or egocentric ear angles). These precise spatial locations relative to other facial regions are captured in the 3D geometric features. Recording the motion of both individual facial regions and their spatial and temporal relationship to the whole face is meaningful, given that the building blocks of facial movement—compartments of facial musculature and the brainstem nuclei that directly control them—are highly topographically arranged^13,14^.

The proof-of-principle work demonstrates the utility of Cheese3D in detecting and characterizing both subtle movements as well as significant and temporally variable movements. Our analysis revealed informative synchronous facial movement patterns that could be used to infer unseen (internal anatomy and physiological functions) from seen (external synchronized facial motion). In particular, subtle differences between two nearly identical facial movements have the capacity to reflect large internal changes. Through electrical stimulation in the brainstem, we demonstrate the ability of Cheese3D to detect such differences, illustrating its ability to reveal the potential of facial behavior as an informative, high-bandwidth readout of neural activity. Moreover, spatially varied facial responses revealed by patterned stimulation in the brainstem suggest that Cheese3D, coupled with precisely synchronized electrophysiology, can tap into the functional organization of facial control machineries, adding to existing knowledge inferred from anatomical tracing. Indeed, our preliminary findings in brainstem recordings of awake mice identified potential pontine reticular nucleus caudalis neurons correlating with the directional ear movement.

We designed Cheese3D to be accessible to others studying facial movements. We demonstrated that six cameras are ideal to capture whole-face dynamics; however, limited setups can still take advantage of Cheese3D by choosing a subset of viewing angles that prioritize relevant facial regions. Although not in the scope of the current work, the framework described in detail here can be adapted for different strains of mice, in a freely moving setup and for tracking development. We anticipate that the method will enable important discoveries across fields in biology and medicine by allowing for noninvasive readout of moment-to-moment changes in body states in mice. The potential applications of high-resolution, whole-face kinematics data made possible by Cheese3D are vast and are likely to inspire a new era of quantitative studies linking facial movements to changes in brain and body brought on by disease, drug exposure, neural processes or other physiological functions that we would otherwise have limited access to based on external observations.

## Methods

### Mouse

All experiments were performed in compliance with protocols approved by the Institutional Animal Care and Use Committee at Cold Spring Harbor Laboratory (protocol number 22-6). Both female and male 2–14-month-old C57BL/6J mice were used in the experiments. Unless stated otherwise, animals were housed in an inverse light-to-dark cycle with constant temperature (20–22 °C) and humidity (54–59%), and had ad libitum access to water and food.

### Video capture, synchronization and 3D calibration system

During the behavioral experiments, the mouse sits inside a custom 3D-printed tunnel and is head-fixed using a custom-made headpost (see CAD files at https://github.com/Hou-Lab-CSHL/cheese3d). The headpost is inserted into a metal rod and secured using a #2-56 thread, 1/4 inch screw tightened on a threaded hole on the side of the rod (see Extended Data Fig. 1). Six high-speed monochrome cameras (FLIR CM3-U3-13Y3M-CS 1/2” Chameleon 3) are used to record the video data at 100 fps. Based on their location relative to the face, the cameras are labeled left (L), right (R), top left (TL), top right (TR), top center (TC) and bottom center (BC) (see Fig. 1b and Supplementary Table 2). The camera location and orientation are selected such that each facial keypoint is in the focused view of at least two cameras (see Supplementary Table 1 for details). The sync LED, attached to the rod where the headpost is secured, is used as a fixed location on top of the mouse’s head, at the point of head fixation. The angle with respect to the sync LED is measured from a top-down view. The lateral cameras (L, R, TL, TR) are equipped with an 8 mm effective focal length (EFL) *f*/1.4 lens (MVL8M23, Thorlabs), and the center cameras (TC and BC) with a 12 mm EFL *f*/1.4 lens (MVL12M23, Thorlabs). Lenses are connected to the body of the camera through a C-to-CS-mount (03-618 Edmund Optics) and 3D-printed 1.1 mm (L, R, TL, TR cameras) or brass 2 mm spacer rings (TC, BC cameras) (03-633, Edmund Optics) for fine focal adjustment. The face is illuminated using two infrared lamps (CMVision IR30 WideAngle) with a piece of Kimwipe (Kimtech Science) covering the LED surface acting as a light diffuser to minimize glare. Most data were recorded using a Windows 10 desktop workstation with the following specifications: Intel Xeon Processor W-2265 (3.5GHz), 256 GB 8 × 32 GB DDR4 RAM, Nvidia RTX A6000 48GB, NVMe SSDs. For alternate setups (see Extended Data Fig. 3d), we used a commodity hardware laptop.

The six cameras were synchronized using Bonsai (v.2.8.1) and an Arduino Mega 2560 REV3, which sends a start signal to Bonsai through the serial port. Upon receiving the trigger signal, Bonsai begins recording frames from all cameras as well as associated metadata for each frame. This hardware signal ensures that different cameras are synchronized at acquisition time. To verify that the camera frames are synchronized post acquisition, a miniature infrared LED (SML-S13RTT86, Mouser Electronics) is positioned to appear in the field of view of all cameras. As a ground-truth synchronization signal, the LED is on for 10 ms every 10 ± 0.5 s.

Post hoc verification of camera synchronization is accomplished by detecting the frame of the rising edge of the LED signal in all views. Using the BC view as a reference, we linearly regress the rising edge times from another view onto the times from the reference view. A perfectly aligned pair of videos should regress onto the identity line, whereas a misaligned view will have a non-zero offset and non-identity slope. The non-zero offset is used as the frame shift to trim the non-reference video, and the slope is used to scale the effective frame rate to match the reference frame rate. This process is repeated systematically for all views and verified using the ground-truth synchronization LED signal.

Video-electrophysiology synchronization is conducted in a similar fashion, with the same synchronization LED signal split and connected to the electrophysiology hardware.

We calibrate camera views using a manufactured calibration board with a standard ChArUco template imprinted on its surface. A vectorized template for the ChArUco board was created using https://github.com/dogod621/OpenCVMarkerPrinter. The template used is for a 7 × 7 ChArUco board (4.5 mm marker length, 6 mm square side length, ArUco dictionary DICT_4 × 4_50). Before and after recording any experimental data, an experimenter held and rotated the ChArUco board in the focused view of all cameras for at least 1 min. These calibration videos were used in Anipose to calibrate the pipeline for triangulation using the default calibration settings.

### Neural network keypoint detections and validations

We used video data from across all mice and experimental conditions (feeding experiments, awake recordings from the anesthesia experiment, recordings from the structure experiment, and so on) to train a single DLC model to track 2D keypoints. Three different DLC models were used throughout our analysis, except when noted otherwise. Differences between models are a result of dataset selection and training procedure (detailed below). For all models, random uniform sampling is used to separate 5% of the frames for testing, while the remaining 95% are used to train the model. Following the standard guidelines provided by DLC, we selected the built-in ResNet-50 model architecture and image augmentation pipeline for our training procedure. Model-specific descriptions are as follows:

- For Figs. 1, 2, 3a–f, 4a–k, a total of 491 frames were selected using the *k*-means clustering algorithm for frame extraction provided by DLC, as well as manually (136 frames were manually taken from the feeding experiment). The model is trained for 1,030,000 iterations using a learning rate schedule of 0.005 for 10,000 iterations, 0.02 for 420,000 iterations, 0.002 for 300,000 iterations and 0.001 for 300,000 iterations. After training, the train set error was 2.16 pixels, and the test set error was 4.6 pixels.
- For Fig. 5a–f, a total of 1,169 frames were selected using the *k*-means clustering algorithm for frame extraction provided by DLC. The model is trained for 1,630,000 iterations using a learning rate schedule of 0.005 for 10,000 iterations, 0.02 for 420,000 iterations, 0.002 for 300,000 iterations and 0.001 for 800,000 iterations. After training, the train set error was 3.3 pixels, and the test set error was 3.95 pixels.
- For Figs. 3g–l, 4l–o, 5i–k, a total of 963 frames were selected using the *k*-means clustering algorithm for frame extraction provided by DLC, as well as manually (136 frames were manually taken from the feeding experiment). The model is trained for 4,700,000 iterations using a learning rate schedule of 0.005 for 100,000 iterations, 0.02 for 2,000,000 iterations, 0.002 for 1,000,000 iterations and 0.001 for 1,700,000 iterations. After training, the train set error was 1.92 pixels, and the test set error was 2.77 pixels.

Back-to-back 3D scanner and Cheese3D recordings in anesthetized mice were used to measure the spatial accuracy and resolution of keypoint detection (see Fig. 1 and Extended Data Fig. 2). Each mouse underwent intraperitoneal injection of ketamine (100 mg kg^−1^) and xylazine (10 mg kg^−1^) cocktail to induce anesthesia, and was scanned first on the 3D scanner (Einscan-SP, SHINING 3D) and then immediately on the Cheese3D setup.

To test the robustness of Cheese3D in detecting 3D keypoints, eight alternative setups were constructed by omitting either two or four cameras of the Cheese3D rig (Extended Data Fig. 3b,c for two examples), or varying camera positions and angles (Extended Data Fig. 3d). The four cameras composing the setup of Extended Data Fig. 3d were equipped with an 8 mm EFL *f*/1.4 lens (MVL8M23, Thorlabs), a C-to-CS-mount adaptor (03-618 Edmund Optics) and 3D-printed 1.1 mm spacer ring. Extended Data Fig. 3e compiles all setup variations, in which excluded cameras are marked with a dot. The design of these setups was based on an informed selection of cameras that could preserve the redundancy of as many keypoints as possible. For each setup, a new 2D model was trained from scratch, using only the labeled frames from the available views. Facial features were extracted and compared against the Cheese3D setup by computing the root mean square error, and the results are summarized in Extended Data Fig. 3e. Dotted squares indicate facial features that could not be calculated because of camera limitations (as some parts of the face were never seen) and were therefore discarded from analysis.

### Triangulation and 3D tracking optimization

We use the trained DLC model to track keypoints in videos for each camera view separately for each experiment. No post-processing is applied to the tracked keypoints. Anipose is used to triangulate 2D keypoints from multiple cameras into a single 3D keypoint per frame using acquired calibration data (see ‘Video capture, synchronization and 3D calibration system’). Next, Anipose optimizes the 3D keypoint tracking for the full recording by reprojecting the 3D keypoints to 2D in each camera view and minimizing the mean squared error of the reprojected points. Concurrently, the frame-to-frame speed of the 3D keypoints is minimized to prevent spurious tracking errors. No post-processing or filtering is applied to the optimized 3D keypoints. To evaluate the performance of the tracking pipeline, we overlaid the optimized 3D keypoints reprojected onto each camera view, and an experimenter curated the accuracy and precision of the tracking results.

### Comparison to an existing facial motion detection system

Recorded videos (640 × 512 pixels) were also tested with Facemap. A region of interest in each video was manually cropped using FFMPEG to match Facemap’s field of view. Although all views were given to Facemap, only the lateral cameras (R, L, TR, TL) resemble the canonical Facemap views. Keypoints were labeled using Cheese3D guidelines. Therefore, Facemap’s keypoints to track the right and left nostrils, nose bridge and paw were not implemented. Ears were not labeled, as Facemap’s base model does not include them. Facemap’s vibrissae keypoints, located at the base of three whiskers, were used to label Cheese3D whisker pad landmarks.

Visual inspection of the output tracking quality by several authors determined the need to fine-tune the Facemap model. For fine-tuning, two to ten frames per view and per experiment were selected using Facemap’s frame selection graphical user interface, resulting in a total of 166 frames. After the first iteration of refinement, some recordings still did not meet the team’s criteria for quality tracking (that is, assessed by visual inspection and jitter speed two orders of magnitude larger than Cheese3D’s), and additional fine-tuning was deemed necessary. For this second iteration, different fine-tuning branches of the same refined model were generated on a mouse basis using two frames per view. For the midline views (TC and BC) without any Facemap analog, an additional iteration of fine-tuning was sometimes required. We took the motionless periods (see ‘Analysis of kinematics during anesthesia’ for more information) to quantify 2D jitter. To make the outputs comparable, we reprojected the 3D output of Cheese3D into the six 2D views. We compared the average speed of the keypoints from each facial region (for example, eyes, nose) across views per mouse.

### Anatomical-based interpretable feature selection

Features are selected and calculated in five tiers with increasing spatial dimension. First, 3D facial keypoints (see Fig. 1 and Extended Data Fig. 2) are selected based on the following criteria: they should be unambiguously and correctly pinpointed by at least three experimenters independently; for the purpose of 3D calibration, be in a focused view by at least two cameras; and reflect natural facial features and anatomy. Second, Euclidean distances between 3D keypoints within a localized facial region (for example, the left eye) are calculated. Third, areas are calculated for the sets of keypoints that form a closed polygon; these include the eye, ear and mouth areas. Fourth, the angle between the ear and snout is calculated as a measure for how forward-orienting the ears are with respect to the whole face. Fifth, the volumes of the nose bulge and whisker pad bulge are calculated to reflect anatomically relevant volumes^5^.

The areas of the eye and ear groups are calculated based on a flattened 2D ellipse. Each group consists of four points defining the major and minor axis endpoints of the ellipse. Given that all four points are not necessarily coplanar, we assume that the ellipse can be bent along the minor axis. To compute the area of this bent ellipse, we begin by defining the major axis (using the front and back of the eye or the base and tip of the ear). Next, we compute the midpoint of the major axis and calculate the Euclidean distance from this midpoint to each of the remaining two minor axis endpoints. The sum of these two distances defines the length of the minor axis after a potential bend has been flattened. Using the major and minor axis lengths, we compute the final ellipse area as the standard area of a 2D ellipse in Euclidean space. The area of the mouth can be computed as the standard area of a triangle in Euclidean space. The right and left upper lip points and one central lower lip point form the vertices of the triangle. The volume of the nose bulge is calculated as an irregular tetrahedron defined by the nose top, left and right pad top and the midpoint between the front of the eyes. We use the standard volume for an irregular tetrahedron in Euclidean space. The volume of the whisker pad bulge is calculated as an irregular pyramid defined by the nose bottom, left and right pad top, and left and right pad side points. We compute the convex hull defined by these points, then calculate the volume of the hull by dividing the hull into smaller tetrahedrons. The specific choice of tetrahedrons used is determined by the SciPy library (v.1.10.1). For a summary of the 17 facial features, see Supplementary Table 3.

### Analysis of keypoint jitter

We quantified the tracking jitter of 3D keypoints and facial features using a 5 min video segment in which the experimenter identified no discernible movement (referred to as the ‘motionless period’). Next, we calculated the magnitude of the frame-to-frame speed of each keypoint during the selected periods that we refer to as the jitter speed of a keypoint. We use frame-to-frame speed as our metric for jitter so that we focus on short-time-scale noise in the tracking instead of slow-moving trends in the tracking that may occur over minutes or hours. To visualize the distribution of keypoint jitter speed in Fig. 2a, we compute a Gaussian kernel density estimate using the histplot function in the Seaborn plotting library (v.0.13.2). The bin size is set to 0.05 mm s^−1^, and the kernel density estimate bandwidth is set using the scotts_factor function in the SciPy library (v.1.10.1). We summarize the distribution of jitter speed during the motionless period by computing the average speed over the entire period per mouse in Fig. 2b.

To assess how the jitter speed of keypoints affects our anatomical features, we computed the absolute frame-to-frame speed of each feature during the selected periods, which we refer to as the jitter speed of an anatomical feature. We selected the 99.9th percentile of the anatomical jitter speed distribution per mouse as our motion threshold. Any movement with a frame-to-frame speed below this threshold will be considered noise. The motion threshold across mice is summarized in Extended Data Fig. 4.

### Headpost design and surgery

The custom-designed stainless steel headpost for head fixation consists of a 6 × 4 × 1 mm rectangular base and a small 10 ×3 mm post that fits into the headpost holder. A groove was added on each lateral end of the base design to facilitate metabond adhesion during implant surgery. The headpost has a conical notch etched on the side to secure in the headpost holder with a screw fastener. The headpost holder is angled at 27.9^∘^, following observation of the natural head angle of the mouse eating to maximize comfort.

To implant the headpost, 2-month-old mice were anesthetized with isoflurane (SomnoFlo, Kent Scientific; 3–5% induction, 1–2% maintenance). Once anesthetic depth was achieved, mice were placed onto a stereotaxic apparatus where body temperature was maintained using a heating pad. After flattening the skull using skull landmarks, the base of the headpost is positioned above the medial-lateral midline and immediately anterior to lambda, and then secured using adhesive cement (Metabond, C&B). Following surgery, animals were administered buprenorphine (0.1 mg kg^−1^) and allowed to recover on a heating pad before returning to their home cages, in which they continued to recover for 1 week before being acclimated to sitting in a tunnel and head fixation for 1–2 weeks.

### EEG surgery and data acquisition

One day before surgery, a biotelemetry unit (HD-X02, Data Sciences International) was thoroughly disinfected in CIDEX OPA solution (Advanced Sterilization Products). Surgery was performed as described above, with two stainless steel screws (00-96 × 1/16, EEG, IROX screw, Data Sciences International) inserted through craniotomy as cortical electrodes (screw 1: 1.5 mm anterior of bregma, 1.5 mm lateral of midline to the left; screw 2: 1.5 mm posterior of bregma, 3.5 mm lateral of midline, contralateral to screw 1. An incision was made to expose the upper trapezius muscles on the animal’s back to place the transmitter into the subcutaneous pocket. A pair of EEG leads was attached to the cortical screws and secured with a small amount of dental cement, and a pair of EMG leads was threaded through the upper trapezius muscles (one on each side of the midline) and held in place by 6-0 polyamide sutures. Tissue adhesive (3M Vetbond) was applied to the skull before attaching the headpost (described above). Post-surgery procedures were the same as described above, with at least 2 weeks recovery time before experiments.

Recording was conducted using a wireless recording system (Data Sciences International): transmitters communicated with PhysioTel receivers connected to PhysioTel Matrix MX2 acquisition interface. An Arduino MEGA generated a square signal that was synchronously sent to an infrared LED, visible in the Cheese3D camera system, and the PhysioTel Signal Interface, connected to the MX2 acquisition interface. The acquisition interface communicates with the EEG recording computer running Ponemah 6.5. All hardware devices are configured within Ponemah to record EEG, EMG and LED synchronizing signal at 1 kHz and body temperature at 1 Hz. After the experiment, data were exported to CSV format using NeuroScore 3.3.1 - Build 9317 (Data Sciences International).

### In vivo electrophysiology recording, electrical stimulation surgery and data acquisition

Surgery was conducted as described above. A craniotomy was performed (anterior to bregma 1.5 mm, lateral 1.0 mm) to insert a grounding pin (male connector pin, A-M systems) at a 45° angle, with the tip of the pin pointing in the rostral direction. The skull was sealed with a tissue adhesive (3M Vetbond) before the headgear implant was attached and further secured with dental cement (C&B metabond, Parkell). The rest of the exposed skull was covered with additional dental cement to further secure the headgear in place. Post-surgery procedures were the same as described above, with at least 1 week recovery time before experimental water deprivation to aid acclimation (1–3 weeks) to awake head fixation.

Following acclimation, a 1 mm diameter brainstem cranial window was made above (posterior to lambda 1.8 mm, lateral 1.25 mm). Head-fixed mice were then stimulated under ketamine and xylazine anesthesia for three to five sessions (‘stimulation sessions’), followed by two awake acute recording sessions (‘recording sessions’). The camera configuration differs slightly from other datasets: the lateral and top center cameras (L, R, TL, TR, TC) are equipped with an 8 mm EFL *f*/1.4 lens (MVL8M23, Thorlabs), and the bottom center camera (BC) is equipped with a 12 mm EFL *f*/1.4 lens (MVL12M23, Thorlabs). In the first stimulation session, multiple brainstem locations were probed with a stimulation grid search. In subsequent stimulation sessions, a 32-channel four-shank silicon probe (A4 × 8-5 mm-100-200-177, NeuroNexus Technologies) was inserted at the mapped locations from the first session. Single electrical pulses (2 ms pulse duration, biphasic) were delivered to either the entire probe or specific divisions of individual shanks by Allego software (NeuroNexus Technologies) every 2–5 s. During the recording sessions, a 32-channel single-shank silicon probe with 46–54 kΩ impedance (H7b or H8b, Cambridge NeuroTech) was inserted into the brain region mapped during the previous stim sessions. The probe was coated with lipophilic dyes DiI or DiO (10% w/v) to reveal the probe track post hoc. Recordings began at least 15 min after probe insertion to ensure recording stability. Voltage signals were amplified using an RHD2132 amplifier (Intan Technologies) and acquired at 30 kHz with a NeuroNexus XDAQ ONE system. After the recording, single electrical pulses (0.5–2 μA) were delivered to all sites on the probe to induce facial movements to verify probe placement location.

Initial spike sorting was performed using Kilosort 2.5 with default parameters, followed by manual curation in Phy. Clusters with inter-spike interval violations, low signal-to-noise ratios or low stability through the recording session were excluded from single-unit analyses.

### Analysis of kinematics during anesthesia

For the anesthesia experiments (see Fig. 3), the awake spontaneous movements of the mice were recorded in Cheese3D for 5 min, followed by intraperitoneal injection of a ketamine (100 mg kg^−1^) and xylazine (10 mg kg^−1^) cocktail to induce anesthesia, before returning to Cheese3D to record facial movement during and recovery from anesthesia. Temperature was maintained on a heating pad, and the exact time of injection was recorded. For the anesthesia re-dosing experiments, mice received either half of the original dosage of ketamine + xylazine or the equivalent volume in saline as a control. Around the 30 min mark from the first injection, the rig door was opened, and the mouse received the re-dosing injection while remaining head-fixed. The data points that were recorded 100 s before and after the second injection were excluded from further analysis. The experimenter was blinded to the content of the second injection.

To measure the wakefulness of each mouse during anesthesia, we computed the magnitude of the frame-to-frame speed of each anatomical feature over the entire recording. We labeled each time point as movement if the frame-to-frame speed crosses the previously computed motion threshold, while time points during which the speed is below the threshold are labeled as no movement. Fig. 3a shows an example raster plot of time points labeled as movement for one mouse.

We analyzed the slow drift of the anatomical and EEG features during anesthesia using a moving average of each feature during the entire recording period. The moving average is computed using a 10 s (facial features) or 40 s (EEG features) wide sliding window average. Fig. 3d shows exemplar filtered features for one mouse over the entire recording period. We visualized the filtered features across all mice and selected three representative features across three facial regions: left ear angle, left eye height and nose bulge. We trained a model across mice to predict time since injection using the selected features during anesthesia. Our model input consists of quadratic terms of the feature at the current time point and initial time point (quadratic terms computed using Scikit Learn’s (v.1.7.2) PolynomialFeatures class) as well as a constant bias. We performed a linear regression from our quadratic input terms to the current time since injection using the Lasso class from Scikit Learn (v.1.7.2), in which we used leave-one-out cross-validation and grid search (over 100 values from 0.1 to 100) to find the optimal regularization coefficient. A separate model was trained for features from the facial regions and the EEG frequency band powers. From a total of 12 sessions (across *n* = 3 mice), we held out three random sessions for testing and used nine for training, repeating this procedure 220 times. We assessed the performance of each model by predicting the time since injection for each test session. A moving-average-filtered (using the same filter as Fig. 3c,d) prediction for a single mouse and exemplar feature set is shown in Fig. 3e. We computed the average root mean squared error of the three sessions for each of the 220 runs in Fig. 3f.

For Fig. 3h,i and Extended Data Fig. 7, we trained models to predict each EEG frequency band power, using the three facial features of left ear angle, left eye height and nose bulge. All models are trained as a linear fit from facial features and past EEG frequency band power signal to current EEG frequency band power (and facial features) signal. To remain agnostic to the necessary features required for prediction, we trained several model classes where differences consist solely of variations on the features passed in and targets predicted out. These are illustrated in detail in Fig. 3h and Extended Data Fig. 7a, but we describe them in brief here. Input facial features consist of the three features during a sliding window of the past 1 min. Input autoregressive EEG features consist of the specific EEG frequency band power during a sliding window of the past 1 min. Additionally, for the time-latent model, the current time since injection is used as an exogenous input feature. The targets are either the current EEG frequency band power signal, current three facial features and the EEG frequency band power signal or the current time since injection. We trained four model classes:

- Linear sliding window, which uses the sliding window facial features as input and current EEG frequency band power as target;
- Linear autoregressive, which uses the sliding window facial features and autoregressive window of the EEG frequency band power as input and the current EEG frequency band power as target;
- Linear vector autoregressive, which uses the sliding window facial features and autoregressive window of the EEG frequency band power as input and the current facial features and the EEG frequency band power as target;
- Time-latent linear model, which trains two models: one uses the sliding window facial features as input and current time since injection as target, and the other uses the current time since injection and an autoregressive window of the EEG frequency band power as input and current EEG frequency band power as target.

For every model class, we train a different model for each mouse and EEG frequency band, using *k*-fold cross-validation across the recordings per mouse (using Scikit Learn’s model_selection.KFold class). We report results on the test folds only. In the case of the time-latent linear model, we train the two sub-models separately, then use the predicted time since injection from the first model as input to the second model, only for testing purposes. For visualization and measuring variance explained in Fig. 3h,i, we smooth the true and predicted EEG frequency band power using a moving average filter with a window of approximately 200 ms. We also report the same results on the unsmoothed data in Extended Data Fig. 7c,d.

For the anesthesia re-dosing experiment, to quantify the difference in facial features between the ketamine + xylazine and control saline solution, facial features were smoothed using a moving average filter with a window of 10 s and *z*-scored. We computed the cumulative running variance using a 6 s window. Then, we took the mean over a 5 min window and computed the difference 15 min before and after the second injection (see Fig. 3l).

### Analysis of chewing kinematics

FED3 (ref. ^59^) was used to dispense chocolate-flavored 20 mg pellets (Dustless Precision Pellets, F05301, Bio-Serv) on demand during the feeding experiment (see Fig. 4). A funnel and tubing were placed underneath the FED3 spout to collect the dispensed pellet and deposit it on a translucent plastic spoon (Measuring Scoop S378, Parkell). The spoon was attached to a servo motor connected to a 3D-printed linear actuator to bring the pellet to the mouth, and then retracted to await the next pellet. Animals in the feeding experiments were gently food-restricted and acclimated for 2 days to eating from the spoon while head-fixed, to facilitate food consumption during the experiment. Each mouse was recorded eating 10 to 13 pellets in one session, and allocated 30 s per pellet. Dropped pellets were excluded from subsequent analysis. For Fig. 4l–o and Extended Data Fig. 9, mice received 50 pellets; dropped pellets were not excluded.

We distinguished the ingestion and mastication phases of chewing based on the shape of the lower envelope of the mouth area during the consumption of each pellet per mouse. An example lower envelope is shown in Fig. 4c. To compute the envelope, we invert the mouth area by negating it, then identifying the peaks of the negated signal using the find_peaks function in SciPy (v.1.10.1) with a window of 200 ms. The lower envelope is defined by linearly interpolating the calculated peaks, then median filtering the interpolated curve with a window of 1.49 s. We defined the transition time from ingestion to mastication as when the lower envelope drops sharply, as shown in Fig. 4c. To quantify the time when the envelope drops, we computed the cumulative area under the envelope during the consumption of each pellet. The cumulative area quickly increases during ingestion, then sharply transitions to a slower increase during mastication. The ‘knee’ in the cumulative area under the envelope was used to quantitatively define the transition time. We used the Kneedle algorithm (with the sensitivity parameter set to 1) to identify the knee point (transition time) for each pellet per mouse shown in Fig. 4d. The Python kneed (v.0.8.5) library was used as our Kneedle implementation.

In Fig. 4e,f, we compared the mouth area and nose bulge during the consumption of pellets by *z*-scoring each anatomical feature separately per pellet per mouse. For Fig. 4f, we plot the normalized mouth area and nose bulge against each other for an example mouse, in which each point constitutes a single frame. We color each point based on whether it occurs before or after the transition time for the corresponding pellet.

We defined the eye protrusion in Fig. 4h–k as the *z*-coordinate of the left eye back keypoint (we observed similar behavior for the right eye back). To quantify the degree of coordination between the mouth area and eye protrusion, we *z*-scored each feature per pellet per mouse. Next, we computed the cross-correlation between the normalized features per pellet per mouse separately for the ingestion and mastication phases. Fig. 4i shows the mean cross-correlation taken across pellets for a single mouse. We identified the peak cross-correlation by selecting the time point with the largest absolute cross-correlation per pellet per mouse, as shown in Fig. 4j,k.

In Fig. 4m–o and Extended Data Fig. 9, the ingestion-related mouth opening was determined as the first moment when the mouth area crossed over a 3 mm^2^ threshold within the first 2 s after spoon dispatch. When no value was found, usually when the pellet was not consumed, the largest possible value (that is, 2 s) was assigned. For Fig. 4m and Extended Data Fig. 9, a line was fitted to each mouse and session using the linregress function in SciPy (v.1.10.1). The mean slope resulting from the regressed lines per mouse was used to compute a two-sided Wilcoxon unpaired test, and the *P* value is reported in Fig. 4n. For Fig. 4o, mouse weight was calculated at the beginning of each session as a proportion of the original weight before food restriction.

### Analysis of in vivo electrical stimulation and electrophysiological recording

Following data collection as described in ‘In vivo electrophysiology recording and electrical stimulation surgery and data acquisition’, video and ephys data were synchronized as described in ‘Video capture, synchronization, and 3D calibration system’. To obtain stimulus-triggered facial responses in Fig. 5b–f, a stimulus trigger signal aligning with the pulse duration was recorded on an analog channel of the electrophysiology data and used to drive an LED visible in the video data. For all but one of the sessions, the rising edge of the recorded trigger signal in the electrophysiological data was used as the time point for stimulus-triggered alignment. In one session, the analog channel was not available because of hardware failures, and the LED in the video was used instead.

Next, we computed the peak displacement of various facial features from their pre-stimulation baseline value. For 100 ms before the stimulation trigger, we measure the average value of a facial feature, then we define the peak displacement as the maximum (positive or negative) deviation away from the baseline average during a 100 ms period after the stimulation trigger. This is done on a trial-by-trial basis for each stimulation. In Fig. 5b (top right), we plotted the absolute value of the peak displacement for the eye height, taking the mean and standard deviation across trials of the same stimulation current amplitude.

We also evaluated our ability to distinguish stimulation-triggered movement from noise using the receiver operating characteristic (ROC) curve in Fig. 5b (bottom right). For a given stimulation current, taking a 100 ms period before stimulation as a ‘false positive’ response (noise) period and 100 ms after stimulation as a ‘true positive’ response (stimulation-triggered movement), we measure detected responses whenever the minimum ipsilateral eye height during the period was below a threshold. Sweeping the threshold from 10% below the minimum to 10% above the maximum eye height, we quantify the response rate during each period as the false positive rate and true positive rate for every threshold. From these data, we construct an ROC curve and measure the area under the curve (AUC) using NumPy’s trapz function. The ROC-AUC for each amplitude is shown in Fig. 5b.

In Fig. 5d, we show the absolute peak displacement of several facial features at 10 μA across all stimulation locations. We omitted some locations (appearing as white blank squares in the figure) when the peak displacement amplitude was below the jitter threshold for that feature, as defined in ‘Analysis of keypoint jitter’ and shown in Extended Data Fig. 5.

For in vivo electrophysiological recordings, spike trains were shuffled 1,000 times using a cyclic shuffling method, in which the entire spike train was shifted by a time between −60 s to −15 s or 15 s to 60 s^60^, with shifted spikes outside the time boundary wrapped to the start or the end of the time duration. To examine the correlation between neural activity and facial movements, actual or shuffled neural firing rates binned at 10 ms were cross-correlated with each facial feature. If the peak of the cross-correlation lies outside the 99.9% CI of the shuffled cross-correlation, and the corresponding lag is within −100 ms to 0 ms, the unit is considered statistically correlated with the facial feature (*P* < 0.001 as set by 1,000 shuffles). The significance of facial feature tuning was further assessed using 95% CIs of shuffled tuning curves and by manually verifying tuning specificity through facial video recordings overlaid with spike sounds.

To determine the predictability of neural activity from facial feature data, we trained a series of Poisson GLMs, also known as linear-nonlinear-Poisson models^61^. For all models, we binned spike trains into 10 ms spike count bins. On a per-recording basis, we trained a model for each facial feature and neural unit pair, in which a model receives a time window of facial features (corresponding to ±750 ms around the current time) to predict the current spike count. For each facial feature or unit pair, we train a model using tenfold cross-validation. To determine the folds, we take the full time range over a recording and partition the time into non-overlapping 10 s windows. A fold is a random sample of 10% of these windows (done using Scikit Learn’s model_selection.KFold class). Input feature windows that extend past the fold are discarded, and those time points are not used for training. Finally, the selected training data are used to fit a Poisson GLM with an intercept and *L*_2_ regularization with a strength of 1 × 10^−8^ (using Neural ModelS’s nemos.glm.PopulationGLM class). The solver used to perform fitting is a gradient descent method with a fixed step size of 1 × 10^−2^. For all figures, we report the prediction only on the test folds. Variance explained in Fig. 5j,k is computed as the mean squared difference between the predicted and true binned firing rate, smoothed by a 200 ms moving average filter. In addition to a cross-validated control, we also perform a negative control on randomized data. Specifically, we repeat the procedure described above 1,000 times, whereby each repetition uses chunk shuffled spike times (before binning) with 50 ms chunks.

### Statistics and reproducibility

Required sample sizes were estimated based on previous publications and experience. The numbers of biological replicates, sessions and animals are reported in the figure legends. No data were excluded from analyses except for the following: for Fig. 1i, mouth area measurements were excluded owing to the lack of ground-truth measurements from the 3D scanner, as a result of the mouse’s orientation with respect to the projector. For Fig. 3j,l, ±100 s of data around the time of the second injection were excluded because the wireless receiver for the EEG was mounted on the rig door, and as such, opening the rig door for injection resulted in unreliable wireless communication during these periods. For Fig. 5g–k, clusters that did not pass spike sorting quality control (inter-spike interval violations, low signal-to-noise ratios or low stability through the recording session) were excluded from single-unit analyses.

Animals were randomly assigned to experimental groups where applicable for data presented in Fig. 3j,k,l; experimenters were blinded when necessary in Figs. 1, 3j–l and Extended Data Fig. 2. For the re-dosing experiment presented in Fig. 3j–l, the experimenters were blinded to the solution that was delivered to each group. The order of group assignment was randomly permuted for each animal. Specifically, bulk solutions for anesthetic and saline were prepared, and an external party blinded the injection solution independently for each session. The identities of all injections were not unblinded until all data analyses were complete, and the authors were unblinded only for producing the final figures. For comparison of Cheese3D against 3D scanner measurement in Fig. 1 and Extended Data Fig. 2, an experimenter obtained 3D positions for facial keypoints using Cheese3D, while an external party annotated 3D positions for the keypoints on the 3D scanner measurements following the same guidelines. The experimental and the third party obtained keypoints independently, blinded to each other’s selections, and the facial feature metrics were quantified and analyzed using automated pipelines applied identically across both sets of annotations, which were revealed to the experimenter when producing the final figures.

### Reporting summary

Further information on research design is available in the Nature Portfolio Reporting Summary linked to this article.

## Online content

Any methods, additional references, Nature Portfolio reporting summaries, source data, extended data, supplementary information, acknowledgements, peer review information; details of author contributions and competing interests; and statements of data and code availability are available at 10.1038/s41593-026-02262-8.

## Supplementary information

Supplementary Information Supplementary Tables 1–4

Reporting Summary

Supplementary Video 1 Demonstration of Cheese3D interactive data visualizer

Supplementary Video 2 Cheese3D tracks whole-face movement in mouse

Supplementary Video 3 Facial keypoints from Cheese3D model overlaid on mesh from 3D scanner

Supplementary Video 4 Example of gradual change in eye height and ear angle during anesthesia (sped up 500×)

Supplementary Video 5 Example transition from ingestion (using incisors) to mastication (using molars) as capitulated in mouth opening area in 3D (slowed down 4×)

Supplementary Video 6 Example eye protrusion during chewing (slowed down 2×)

## Source data

Source Data Statistical Source Data for Figs. 1–5, Extended Data Figs. 2–10

## Figures and Tables

**Extended Data Fig. 8:**
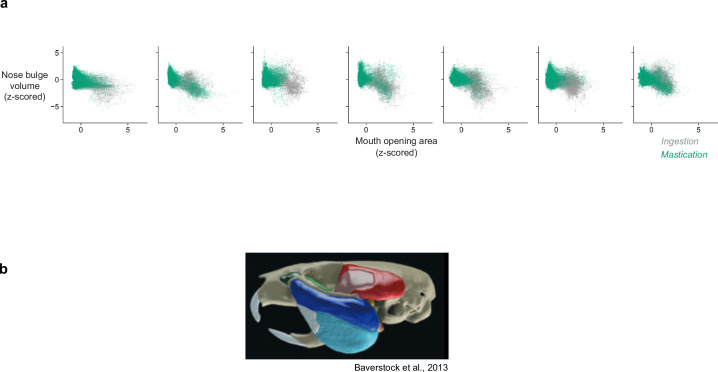
Visualizing putative division between ingestion and mastication across mice. (**a**)Mouth opening area and nose bulge volume (z-scored) scatter plot across mice, sorted by ascending putative transition time between ingestion (gray) and mastication (green) (see Fig. 4d). The example mouse shown in Fig. 4f is second from the right. (**b**)Segmentation of muscles of mastication shown wrapping around the eye socket^26^. Source data.

## Data Availability

All raw data used for analyses in the paper are publicly available on Zenodo at 10.5281/zenodo.18508087 (ref. ^62^). Source data are provided with this paper.

## Code availability

Cheese3D software and example datasets are available at https://github.com/Hou-Lab-CSHL/cheese3d. Additionally, a copy of the analysis code is available on Zenodo at 10.5281/zenodo.18573618 (ref. ^63^).
