# Supplementary material for: Cheese3D enables sensitive detection and analysis of whole-face movement in mice
Source: Nat Neurosci. 2026 Apr 27;29(6):1510–21. doi: 10.1038/s41593-026-02262-8 (PMC13246446; doi:10.1038/s41593-026-02262-8)
Supplement: Supplementary file 1 — Supplementary Tables 1–4 [file 41593_2026_2262_MOESM1_ESM.pdf]

nature neuroscience

Supplementary information https://doi.org/10.1038/s41593-026-02262-8

Cheese3D enables sensitive detection and analysis of whole-face movement in mice

In the format provided by the authors and unedited

Supplementary Information

Supplementary Tables

Facial keypoint	Left	Right	Top Left	Top Right	Top Center	Bottom Center
nose(bottom)	Y	Y				Y
nose(tip)	Y	Y	Y	Y	Y	Y
nose(top)	Y	Y	Y	Y	Y	Y
pad(top)(left)	Y		Y			Y
pad(side)(left)	Y					Y
pad(top)(right)		Y		Y		Y
pad(side)(right)		Y				Y
pad(center)	Y	Y				Y
lowerlip	Y	Y				Y
upperlip(left)	Y					Y
upperlip(right)		Y				Y
eye(front)(left)	Y		Y		Y	
eye(top)(left)	Y		Y		Y	
eye(back)(left)	Y		Y		Y	
eye(bottom)(left)	Y		Y		Y	
eye(front)(right)		Y		Y	Y	
eye(top)(right)		Y		Y	Y	
eye(back)(right)		Y		Y	Y	
eye(bottom)(right)		Y		Y	Y	
ear(base)(left)	Y		Y			
ear(top)(left)	Y		Y			
ear(tip)(left)	Y		Y			
ear(bottom)(left)	Y		Y			
ear(base)(right)		Y		Y		
ear(top)(right)		Y		Y		
ear(tip)(right)		Y		Y		
ear(bottom)(right)		Y		Y		

Supplementary Table 1: Keypoints labeled per camera view

Camera	Distance from Sync LED	Angle w.r.t. Sync LED	Angle w.r.t Rig Floor
Bottom Center	14 cm	$90°$	$-15°$
Top Center	13 cm	$90°$	$15°$
Left	11 cm	$180°$	$0°$
Right	11 cm	$0°$	$0°$
Top Left	10 cm	$120°$	$10°$
Top Right	10 cm	$60°$	$10°$

Supplementary Table 2: Camera distances and angles in the Cheese3D behavioral setup.

Feature	Definition
Ear width left	Euclidian distance between the top and bottom keypoints of the left ear. Approximating the contour of the pinna as two arcs, one dorsal and one ventral, ear top and bottom are defined as the points of maximum curvature of each arc.
Ear width right	Euclidian distance between the top and bottom keypoints of the right ear. See ear width left for a definition of ear top and bottom keypoints.
Ear height left	Euclidian distance between the base and tip keypoints of the left ear. The tip of the ear is defined as the most caudal point of the pinna contour, usually coinciding with the intersection between the dorsal and ventral arcs, as defined in ear width left. The base keypoint lies on the intertragal notch.
Ear height right	Euclidian distance between the base and tip keypoints of the right ear. See ear height left for a definition of ear base and tip keypoints.
Eye width left	Euclidian distance between the front and back keypoints of the left eye, corresponding to the medial and lateral commissures.
Eye width right	Euclidian distance between the front and back keypoints of the right eye. See eye width left for a definition of these keypoints.
Eye height left	Euclidian distance between the top and bottom keypoints of the left eye. Approximating the contour of the eye as two arcs, that outline the upper and lower eyelids, top and bottom of the eye are defined as the points of maximum curvature of each arc.
Eye height right	Euclidian distance between the top and bottom keypoints of the right eye. See eye height left for a definition of ear top and bottom keypoints.
Ear area left	Elliptical area defined by the top, tip, base and bottom keypoints of the left ear as the endpoints of the major and minor axes. See ear width left and ear height left for definitions of the ear keypoints.
Ear area right	Elliptical area defined by the top, tip, base and bottom keypoints of the right ear as the endpoints of the major and minor axes. See ear width left and ear height left for definitions of the ear keypoints.
Eye area left	Elliptical area defined by the front, top, back and bottom keypoints of the left eye as the endpoints of the major and minor axes. See eye width left and eye height left for definitions of the eye keypoints.
Eye area right	Elliptical area defined by the front, top, back and bottom keypoints of the right eye as the endpoints of the major and minor axes. See eye width left and eye height left for definitions of the eye keypoints.
Mouth area	Triangle area defined by the center of the lower lip and the right/left commissures tangent to the upper lip.
Nose bulge volume	Volume of the convex hull enclosed by the left and right upper whisker pad points, top of the nose, and the middlepoint between the front keypoints of the eye. Nose top is the most caudal point on the nose, usually located along the nasal bone axis, and visually identified by a change in fur length. The whisker pad points correspond to the most posterior points along the upper border of the whisker pad.
Cheek bulge volume	Volume of the convex hull enclosed by the whisker pad points, i.e., upper and lateral borders of right and left whisker pads and the bottom of the nose. The bottom of the nose is the midpoint between both nostrils.
Ear angle left	Three-dimensional angle calculated between base to tip of the left ear and base of the left ear to whisker pad center, i.e., the philtrum middlepoint.
Ear angle right	Same as ear angle left for the right ear.

Supplementary Table 3: Facial feature and keypoint definitions

Location	Current (μA)	Ipsilateral eye height (μm)	Ipsilateral ear angle (°)	Nose bulge volume (mm^3)	Whisker pad volume (mm^3)
1U	1.0	21.33 ± 4.51	0.35 ± 0.14	0.15 ± 0.06	1.82 ± 0.52
1U	2.0	14.16 ± 6.77	0.33 ± 0.18	0.10 ± 0.06	0.98 ± 0.82
1U	5.0	45.20 ± 10.05	2.44 ± 0.80	0.40 ± 0.20	2.43 ± 0.78
1U	10.0	71.20 ± 12.65	2.17 ± 0.58	0.57 ± 0.13	3.42 ± 0.75
2U	1.0	19.63 ± 5.31	0.40 ± 0.18	0.24 ± 0.07	1.23 ± 0.84
2U	2.0	18.51 ± 6.88	0.60 ± 0.35	0.19 ± 0.08	1.06 ± 0.88
2U	5.0	31.24 ± 7.71	1.12 ± 0.39	0.29 ± 0.07	2.72 ± 0.68
2U	10.0	80.88 ± 18.94	1.94 ± 0.52	0.71 ± 0.14	4.99 ± 0.69
3U	1.0	13.90 ± 4.89	0.42 ± 0.20	0.19 ± 0.08	1.18 ± 0.90
3U	2.0	9.17 ± 3.79	0.38 ± 0.11	0.19 ± 0.11	1.57 ± 1.17
3U	5.0	12.23 ± 8.03	0.59 ± 0.22	0.30 ± 0.11	1.88 ± 0.26
3U	10.0	30.11 ± 7.85	1.36 ± 0.53	0.62 ± 0.09	4.91 ± 0.53
4U	1.0	7.73 ± 4.15	0.85 ± 0.47	0.16 ± 0.04	2.11 ± 0.28
4U	2.0	12.69 ± 9.33	0.71 ± 0.46	0.14 ± 0.05	1.95 ± 0.58
4U	5.0	16.54 ± 8.38	0.30 ± 0.22	0.15 ± 0.06	1.79 ± 0.61
4U	10.0	26.34 ± 10.31	1.20 ± 0.57	0.22 ± 0.08	2.22 ± 0.68
1L	1.0	18.14 ± 4.88	0.39 ± 0.14	0.25 ± 0.06	0.92 ± 0.75
1L	2.0	20.37 ± 6.44	0.33 ± 0.14	0.26 ± 0.06	1.09 ± 0.79
1L	5.0	21.61 ± 7.05	0.37 ± 0.12	0.25 ± 0.07	1.07 ± 0.85
1L	10.0	32.36 ± 9.71	1.08 ± 0.37	0.44 ± 0.14	3.92 ± 0.98
2L	1.0	21.82 ± 6.75	0.45 ± 0.22	0.11 ± 0.04	0.39 ± 0.43
2L	2.0	12.57 ± 6.29	0.46 ± 0.20	0.13 ± 0.06	0.73 ± 0.79
2L	5.0	11.66 ± 5.75	0.46 ± 0.26	0.12 ± 0.06	0.24 ± 0.09
2L	10.0	20.32 ± 4.84	1.43 ± 0.39	0.59 ± 0.17	4.08 ± 1.05
3L	1.0	10.41 ± 5.46	0.48 ± 0.15	0.12 ± 0.11	0.62 ± 0.87
3L	2.0	10.96 ± 6.91	0.44 ± 0.29	0.08 ± 0.03	0.46 ± 0.40
3L	5.0	14.02 ± 8.75	0.44 ± 0.16	0.08 ± 0.02	0.30 ± 0.10
3L	10.0	5.79 ± 2.62	0.98 ± 0.57	0.15 ± 0.05	1.74 ± 0.75
4L	1.0	19.37 ± 7.52	0.37 ± 0.20	0.16 ± 0.07	1.89 ± 0.75
4L	2.0	21.64 ± 8.79	0.32 ± 0.16	0.16 ± 0.07	1.82 ± 0.69
4L	5.0	21.08 ± 8.52	0.29 ± 0.16	0.17 ± 0.07	1.96 ± 0.73
4L	10.0	21.78 ± 10.64	0.62 ± 0.29	0.18 ± 0.06	1.90 ± 0.33

Supplementary Table 4: Summary statistics (mean $\pm$ std) for **Figure 5e**.
