# Supplementary material for: Cheese3D enables sensitive detection and analysis of whole-face movement in mice
Source: Nat Neurosci. 2026 Apr 27;29(6):1510–21. doi: 10.1038/s41593-026-02262-8 (PMC13246446; doi:10.1038/s41593-026-02262-8)
Supplement: Supplementary file 2 — Reporting Summary [file 41593_2026_2262_MOESM2_ESM.pdf]

nature portfolio

Corresponding author(s): Xun Helen Hou

Last updated by author(s): 2/9/2026

Reporting Summary

Nature Portfolio wishes to improve the reproducibility of the work that we publish. This form provides structure for consistency and transparency in reporting. For further information on Nature Portfolio policies, see our Editorial Policies and the Editorial Policy Checklist.

Statistics

For all statistical analyses, confirm that the following items are present in the figure legend, table legend, main text, or Methods section.

n/a	Confirmed	
☐	☒	The exact sample size (n) for each experimental group/condition, given as a discrete number and unit of measurement
☒	☐	A statement on whether measurements were taken from distinct samples or whether the same sample was measured repeatedly
☐	☒	The statistical test(s) used AND whether they are one- or two-sided<br>*Only common tests should be described solely by name; describe more complex techniques in the Methods section.*
☒	☐	A description of all covariates tested
☐	☒	A description of any assumptions or corrections, such as tests of normality and adjustment for multiple comparisons
☐	☒	A full description of the statistical parameters including central tendency (e.g. means) or other basic estimates (e.g. regression coefficient) AND variation (e.g. standard deviation) or associated estimates of uncertainty (e.g. confidence intervals)
☐	☒	For null hypothesis testing, the test statistic (e.g. F, t, r) with confidence intervals, effect sizes, degrees of freedom and P value noted<br>*Give P values as exact values whenever suitable.*
☒	☐	For Bayesian analysis, information on the choice of priors and Markov chain Monte Carlo settings
☒	☐	For hierarchical and complex designs, identification of the appropriate level for tests and full reporting of outcomes
☐	☒	Estimates of effect sizes (e.g. Cohen's d, Pearson's r), indicating how they were calculated

Our web collection on statistics for biologists contains articles on many of the points above.

Software and code

Policy information about availability of computer code

Data collection	For video data and metadata: Bonsai (v2.8.1); For EEG data: NeuroScore (v3.3.1), Ponemah (v6.5); For electrophysiology data: NeuroNexus Allego (v2025), NeuroNexus Curate (v2023), Kilosort (v2.5), Phy (v2)
Data analysis	For analysis we used Python (v3.10), Scikit Learn (v1.7.2), SciPy (v1.10.1), NumPy (v1.26.4), Pandas (v2.1.4), Jax (v0.4.38), Nemos (v0.2.3). To generate the facial feature data used in analysis, we used DeepLabCut (https://github.com/Hou-Lab-CSHL/DeepLabCut.git), Anipose (https://github.com/Hou-Lab-CSHL/anipose.git, https://github.com/Hou-Lab-CSHL/aniposelib.git) and wrote Cheese3D, whose code is available publicly at https://github.com/Hou-Lab-CSHL/cheese3d. Dependencies of Cheese3D are documented in https://github.com/Hou-Lab-CSHL/cheese3d/blob/main/pixi.lock and https://doi.org/10.5281/zenodo.18573618.

For manuscripts utilizing custom algorithms or software that are central to the research but not yet described in published literature, software must be made available to editors and reviewers. We strongly encourage code deposition in a community repository (e.g. GitHub). See the Nature Portfolio guidelines for submitting code & software for further information.

Data

Policy information about availability of data

All manuscripts must include a data availability statement. This statement should provide the following information, where applicable:

- Accession codes, unique identifiers, or web links for publicly available datasets
- A description of any restrictions on data availability
- For clinical datasets or third party data, please ensure that the statement adheres to our policy

All data will be made publicly available upon publication at https://doi.org/10.5281/zenodo.18508087. Demonstration data is available at https://labshare.cshl.edu/shares/houlab/www-data/cheese3d_paper_data/cheese3d_demo.tar.gz.

Research involving human participants, their data, or biological material

Policy information about studies with human participants or human data. See also policy information about sex, gender (identity/presentation), and sexual orientation and race, ethnicity and racism.

Reporting on sex and gender	No human participants
Reporting on race, ethnicity, or other socially relevant groupings	No human participants
Population characteristics	No human participants
Recruitment	No human participants
Ethics oversight	No human participants

Note that full information on the approval of the study protocol must also be provided in the manuscript.

Field-specific reporting

Please select the one below that is the best fit for your research. If you are not sure, read the appropriate sections before making your selection.

☒ Life sciences ☐ Behavioural & social sciences ☐ Ecological, evolutionary & environmental sciences

For a reference copy of the document with all sections, see nature.com/documents/nr-reporting-summary-flat.pdf

Life sciences study design

All studies must disclose on these points even when the disclosure is negative.

Sample size	We did not compute sample-size analysis. For Figure 1, we used 7 mice. For Figure 2, we used 5 mice. For Figure 3a-f, we used 3 mice. For Figure 3g-i, we used 6 mice. For Figure 3j-l, we used 4 mice. For Figure 4, we used 7 mice. For Figure 5, we used 4 mice. These are sufficiently many mice, in line with similar studies.
Data exclusions	For Figure 1i, mouth area measurements were excluded due to the lack of ground truth measurements from the 3D scanner, as a result of the mouse's orientation with respect to the projector. For Figure 3j-l, +/-100 sec of data around the time of second injection was excluded since wireless communication with the recording device is unreliable during these periods. Additionally, for Figure 3h-l, one mouse died while recording and had fewer samples per subject compared to other mice; thus, it was excluded when the low sample size would impair statistically valid analysis. For Figure 5g-k, clusters with inter-spike interval violations, low signal-to-noise ratios, or low stability through the recording session were excluded from single-unit analyses.
Replication	Experiments were repeated across multiple mice of both sexes and different ages. The exact number of replicates can be found in the Sample size section above. Analysis was successfully replicated using the publicly available code and data.
Randomization	For the anesthesia re-dosing experiment, we randomly assign blinded labels to the prepared injections. This was done by an external third-party randomly at their discretion. For all the experiments, there were not multiple sample groups, all mice underwent every experimental condition.
Blinding	For anesthesia experiments where mice are delivered a second injection containing half the initial dose or saline control solution, the experimenters were blinded to which solution was delivered. Specifically, bulk solutions for anesthetic and control were prepared, and an external party prepared the second injection per subject. The identity of each injection was hidden and masked as "Group A" or "Group B". The solution corresponding to each group was randomly chosen per day. The final identities of all injections were not revealed until all hypotheses and initial data analysis was complete, and the authors were un-blinded only for producing final figures.

Reporting for specific materials, systems and methods

We require information from authors about some types of materials, experimental systems and methods used in many studies. Here, indicate whether each material, system or method listed is relevant to your study. If you are not sure if a list item applies to your research, read the appropriate section before selecting a response.

Materials & experimental systems

n/a	Involved in the study
☒	☐ Antibodies
☒	☐ Eukaryotic cell lines
☒	☐ Palaeontology and archaeology
☐	☒ Animals and other organisms
☒	☐ Clinical data
☒	☐ Dual use research of concern
☒	☐ Plants

Methods

n/a	Involved in the study
☒	☐ ChIP-seq
☒	☐ Flow cytometry
☒	☐ MRI-based neuroimaging

Animals and other research organisms

Policy information about studies involving animals; ARRIVE guidelines recommended for reporting animal research, and Sex and Gender in Research

Laboratory animals	Both female and male C57BL/6J mice 2-14 months of age were used for experiments.
Wild animals	No wild animals involved.
Reporting on sex	All data was aggregated across sex. We did not perform any sex-based analysis, since sex differences were not relevant to our studies.
Field-collected samples	No field samples were collected.
Ethics oversight	All experimental procedures were conducted per IACUC-approved protocols (number 22-6); ethics approval was received from the IACUC board at Cold Spring Harbor Laboratory.

Note that full information on the approval of the study protocol must also be provided in the manuscript.

Plants

Seed stocks	No seed stocks.
Novel plant genotypes	No novel plant genotypes.
Authentication	Not applicable.
